# *De novo* assembly of the sea trout (*Salmo trutta* m. *trutta*) skin transcriptome to identify putative genes involved in the immune response and epidermal mucus secretion

**DOI:** 10.1371/journal.pone.0172282

**Published:** 2017-02-17

**Authors:** Magdalena Malachowicz, Roman Wenne, Artur Burzynski

**Affiliations:** Institute of Oceanology PAS, Sopot, Poland; Institut National de la Recherche Agronomique, FRANCE

## Abstract

In fish, the skin is a multifunctional organ and the first barrier against pathogens. Salmonids differ in their susceptibility to microorganisms due to varied skin morphology and gene expression patterns. The brown trout is a salmonid species with important commercial and ecological value in Europe. However, there is a lack of knowledge regarding the genes involved in the immune response and mucus secretion in the skin of this fish. Thus, we characterized the skin transcriptome of anadromous brown trout using next-generation sequencing (NGS). A total of 1,348,306 filtered reads were obtained and assembled into 75,970 contigs. Of these contigs 48.57% were identified using BLAST tool searches against four public databases. KEGG pathway and Gene Ontology analyses revealed that 13.40% and 34.57% of the annotated transcripts, respectively, represent a variety of biological processes and functions. Among the identified KEGG Orthology categories, the best represented were signal transduction (23.28%) and immune system (8.82%), with a variety of genes involved in immune pathways, implying the differentiation of immune responses in the trout skin. We also identified and transcriptionally characterized 8 types of mucin proteins–the main structural components of the mucosal layer. Moreover, 140 genes involved in mucin synthesis were identified, and 1,119 potential simple sequence repeats (SSRs) were detected in 3,134 transcripts.

## Introduction

Brown trout (*Salmo trutta*) is a salmonid fish species of significant ecological and commercial value that is, naturally distributed throughout all of Europe and in adjacent waters [1]. This species represent two different life strategies: resident and migratory [2]. An anadromous form, called sea trout (*Salmo trutta* m. *trutta*) migrates to the sea for feeding and returns to its natal rivers for spawning [3]. The highest total (commercial and recreational) sea trout catches in the Baltic Sea, above 1000 tons, were recorded in the early and late 1990s. Since then, the total sea trout catches have been decreasing and are now below 500 tons [4]. Several factors have contributed to this decrease, including: pollution, environmental degradation and diseases [5–7]. One of the most common diseases that deplete sea trout populations is furunculosis, which is caused by the gram-negative bacteria *Aeromonas salmonicida* [8]. This disease is transmitted by close contact between infected and healthy fish, it begins with hypertrophy of the epidermis, followed by ulcers spreading into the subcutaneous tissue, and leads to general infection and septicemia. Teleostean fish have evolved innumerable host-pathogen relationships, some of which are unique to only a few or a single species [9]. Even related species can show differences in susceptibility to pathogens due to varied skin morphology [9] and gene activity [10]. Coho salmon (*Oncorhynchus kisutch*) is less susceptible to infectious hematopoietic necrosis virus (IHNV) than rainbow trout (*Oncorhynchus mykiss*) or Atlantic salmon (*Salmo salar*) [11]. Moreover, rainbow trout and Atlantic salmon are more resistant against *A*. *salmonicida*, while brown trout is the most susceptible [12].

For a pathogen such as *A*. *salmonicida* to infect the host, innate barriers must first be crossed, including a shield of scales, skin and mucus [13, 14]. Fish skin traps and washes away pathogens and contains many immune factors, such as: antimicrobial peptides [15, 16], C-reactive proteins [17], complement components [18], immunoglobulins [19], lectins [20], lysozymes [21] and proteases [22]. In addition to fish skin functioning as a mechanical barrier, it is a multi-functional organ (with roles in ion regulation, excretion, ion and thermal regulation) and metabolically active tissue [23–25]. These functions are possible due to the complex structure and cell composition of the skin [23]. The important defensive role of the skin in the innate immune system is well known and has been studied in several fish species [22, 26–29]. However, there is a lack of genomic information such as the skin transcriptome of sea trout.

One of the most distinctive features of fish skin is the production of mucus by the unicellular glands of the epidermis, mainly goblet cells and club cells [28]. Mucus protects the underlying epithelium from chemical, enzymatic and mechanical damage [30]. Bacterial infection or a change in diet can increase mucus secretion and change the glycosylation pattern and thus the bacterial adherence to the skin, potentially increasing pathogen removal [14, 31]. The complexity of glycan epitopes and sialylation seem to be key factors in the host–pathogen interaction between Atlantic salmon and *A*. *salmonicida* [14]. The goblet cells in the skin epidermis are responsible for the production and maintenance of the mucosal layer via the synthesis and secretion of mucins [27, 32]. Mucins are high molecular weight glycoproteins with a large number of tandem repeats that are rich in proline, threonine and serine (the PTS domain) [33]. These PTS regions tends to be poorly conserved [30]. In vertebrates, over 20 mucin types have been characterized thus far. Mucins can be divided into two structurally distinct subfamilies: large secreted gel- forming mucins (SGFM) and membrane-bound mucins [34]. The SGFM subfamily include Muc2, Muc5AC, Muc5B, Muc6, and Muc19. These mucins contain a von Willebrand factor type D (VWD) domain, cysteine-rich C8 (C8) domain and C-terminal cysteine-knot (CK) domain, which are all involved in the oligomerization of mucins [35]. By contrast, membrane-bound mucins (Muc1, Muc3, Muc4, Muc7-9, and Muc12–22) do not oligomerize and are characterized by specific domains such as the C-terminal sea urchin sperm protein-enterokinase-agrin (SEA) domain, an epidermal growth factor (EGF) or EGF-like (EGF-L), and a transmembrane domain (TM) [36]. Mucins constitute an important part of the mucosal defense against infection [37, 38]. Furthermore, based on mucin studies of Atlantic salmon, skin and intestinal mucins have different biochemical characteristics, which in turn affect their binding of the pathogens [14]. The density of the skin mucins was lower and glycans were shorter and less branched [14]. Recently, function of fish mucins was confirmed in few studies. Pérez-Sánchez et al. [39] challenged in the study of sea bream, specific expression of mucins under different nutritional conditions, parasite infections and the tissue-specific expression. Mucin structure and tissue-expression were also studied in carp and zebrafish [40, 41]. Moreover, stressed rainbow trout had greater proportions (two times higher) of goblet cells that contained mucins with sialic acid compared to control [42]. Peatman et al. [43] used utilized high-density Affymetrix microarrays to examine gene expression profiles in channel and blue catfish skin upon *A*. *hydrophila* infection. Several recent studies have described mucin sequences in teleost fish [27, 44] but mucin genes have not been characterized in the transcriptomes of different tissues from brown trout, such as the liver, kidney, gut, gill, embryo, testis, intestine, bones, muscle, heart, brain and ovary [45, 46].

In this study, we assembled and characterized the skin transcriptome of sea trout using the Roche GS-FLX 454 pyrosequencing system. A large number of genes involved in immune reactions and mucus secretion were identified. Moreover, this study is the first to present and characterize trout mucins. The generated data add to the growing sequence database for brown trout and provide the first representation of the trout skin transcriptome and valuable resources for functional genomics.

## Materials and methods

### Ethics statement

This study was performed in accordance with the three Rs for the humane use of animals in scientific research and was approved by the Local Ethics Committee on Animal Experimentation of the Inland Fisheries Institute of Olsztyn, Poland (Nr 51/N/2005).

### RNA extraction and pyrosequencing

Six specimens of sea trout were obtained from the Department of Salmonid Fish Breeding of the Inland Fishery Institute of Rutki, Poland in 2010. Extraction of total RNA from the deep frozen skin tissue upstream the anal fin was performed using a GenElute Mammalian Total RNA Miniprep Kit (Sigma) according to the manufacturer’s protocol, with minor modifications. Homogenization was performed in custom buffer composed of 100 mM Tris, 1.4 M NaCl, 20 mM EDTA, 2% CTAB, proteinase K (20 mg/ml) and 0.03 mM 2-mercaptoethanol. The incubation time was 30 minutes. The DNA was removed following the protocol provided by the manufacturer. The RNA isolate was stored at -20°C.

Total RNA was used for cDNA synthesis using the SMART (Switching Mechanism At 5' end of RNA Template) kit from BD Biosciences Clontech. The cDNA library, was later normalised using the duplex-specific nuclease (DSN) method [47]. Approximately 15 μg of cDNA were used for sequencing (Roche GS-FLX) at the Max Planck Institute for Molecular Genetics (Berlin, Germany), following described procedures [48].

### Assembly and annotation

The raw reads produced from sea trout sequencing were cleaned by removing adapters and low quality sequences. The quality score limit was set to 0.05 and the reads shorter than 50 bp were discarded using CLC Genomics Workbench software (v.7.5.5, CLC Bio, Qiagen, Aarhus, Denmark). The quality reads were assembled into contigs using de-Bruijn graphs in CLC Genomics Workbench. The generated contigs were searched against the National Center of Biotechnology Information non-redundant protein database (NCBI nr), Swiss-Prot (ftp://ftp.ncbi.nlm.nih.gov/blast/db), Clusters of Eukaryotic Orthologous Groups (KOG; ftp://ftp.ncbi.nih.gov/pub/COG/KOG) and the Teleost Ensembl database. The teleost fish database was created by joining reference protein sequences of 11 teleost fish species (Amazon molly—*Poecilia formosa*, cave fish—*Astyanax mexicanus*, cod—*Gadus morhua*, fugu—*Takifugu rubripes*, medaka- *Oryzias latipes*, southern platyfish—*Xiphophorus maculatus*, spotted gar—*Lepisosteus oculatus*, three-spined stickleback—*Gasterosteus aculeatus*, green spotted puffer*—Tetraodon nigroviridis*, Nile tilapia—*Oreochromis niloticus*, zebrafish—*Danio rerio*) obtained from the Ensembl database (http://www.ensembl.org). Annotations were completed using the BLASTX tool implemented in BLAST+ (v.2.2.29) [49], with an E-value cut-off of 10^−5^. A list of the top BLAST hits was obtained using Blast2GO software [50] and was based on the best-aligned results. Sequences with positive matches in the NCBI nr protein database were further searched for Gene Ontology (GO) terms [51] with Blast2GO. The level 2 GO terms were retrieved and classified into three categories: cellular component, biological process and molecular function. Furthermore, Kyoto Encyclopedia of Genes and Genomes (KEGG) pathways were assigned using the single directional best-hit (SBH) method in the KEGG Automatic Annotation Server (KAAS) [52]. This tool assigns KEGG Orthology (KO) identifiers to sequences based on their similarity, which is directly linked to an object in the KEGG pathway map or the BRITE functional hierarchy [52]. Moreover, sequences without significant BLAST hits were searched against the noncoding RNA database using Rfam 12.0 (http://rfam.xfam.org) [53].

The results from this study were also compared to the previously published trout multi-tissue transcriptome from the River Hayle and River Teign in southwest England, which was obtained from the authors [45]. Raw data are available in the Gene Expression Omnibus (GEO) under accession number GSE45637. Contig identification and GO term annotation were completed by same methodology as previously described, and the GO results from both transcriptomes were compared. To find enriched GO terms between skin and other tissues, Fisher’s exact test was performed with Blast2GO, applying a False Discovery Rate (FDR) <0.05 [54]. We also investigated nucleotide-level comparisons by mapping skin transcriptome reads to the multi-tissue transcriptome using CLC Genomics Workbench with default parameters.

### Mucin bioinformatics

Based on the top BLAST hit descriptions, contigs annotated as mucin genes were extracted from skin and multi-tissue trout transcriptomes and used for further analysis. Sequences were checked and confirmed for homologs in the GenBank nr database and *Salmo salar* genome [55], using the program BLASTX and BLASTN (http://blast.ncbi.nlm.nih.gov/Blast.cgi). The coding regions (CDS) of each contig sequence were predicted and translated using GENSCAN (http://genes.mit.edu/GENSCAN.html) [56]. The functional domains and motifs were identified using Simple Modular Architecture Research Tool (SMART) software, which allows the identification and annotation of domain architectures (http://smart.embl-heidelberg.de) [57]. In addition, transmembrane domains (TM) were predicted with TMHMM v. 2.0 software [58]. To further scrutinize I-mucin (I-Muc) genes, a representative reference teleost database was created. All protein sequences described as intestinal-mucin, mucin-5AC, mucin-5B and mucin-2, which are available at the GenBank database (www.ncbi.nlm.nih.gov) [59], were downloaded (771 sequences), including the Baltic cod transcriptome [60]. Next, trout sequences were searched against the created database (E-value cut-off = 10^−10^). Furthermore, all homologous bony fish proteins were analyzed with SMART to identify protein domains. Only sequences with annotated VWD and C8 domains, which are typical for SGFM, were selected, with one representative species per family, and each type of mucin was represented. A total of 15 mucin sequences from six species (five families) were used in the reconstruction of I-Muc relationships, and 18 sequences from four species (three families) were used for phylogenetic analysis of SGFM (Muc5, Muc2 and I-Muc). For phylogenetic analysis, VWD and C8 domain sequences were extracted and aligned using MUSCLE via MEGA v.7.0.14 [61] with default parameters. The best substitution model was chosen using MEGA. The phylogenetic trees were inferred using a maximum likelihood approach with the Whelan and Goldman (WAG) substitution model and gamma-distributed rates. The reliability of the tree was assessed by bootstrapping [62] using 1000 bootstrap replicates. Furthermore, genes involved in mucin synthesis were identified in the sea trout skin transcriptome based on gene name searches against previous studies in mammals and fish [44, 63] and the KEGG mucin type O-glycan biosynthesis pathway.

### SSR detection

Potential microsatellite markers were detected using MSDB software [64]. We identified all SSRs in our data set with a repeat length ranging from 2 to 6 and a minimum sequence length of 12 nt. Mononucleotide repeats were ignored because it was difficult to distinguish them from polyadenylation products.

## Results

### Sequencing and assembly

In this study, 1,440,373 raw reads with an average read length of 333 bp were generated using pyrosequencing. After the low-quality sequences and short reads were trimmed, 1,348,306 (93.61%) high-quality reads were used for *de novo* assembly in CLC Genomics Workbench (Table 1). A total of 75,970 contigs with a mean length of 668 bp were produced (Table 1). The transcriptome data analysis workflow is displayed in S1 Fig. The sea trout raw transcriptome sequences from this study were deposited in the NCBI Sequence Read Archive (SRA) under accession number SRP075946. Assembled contigs and annotation information are available from the authors upon request.

**Table 1:** Statistical summary of sea trout skin transcriptome data.

**Sequencing**	Number of reads	1,440,373
**Assembly**	Total clean reads	1,348,306
	Total bases (bp)	470,264,108
	Total assembled bases (bp)	424,100,862
	Number of contigs	75,970
	Average contig length (bp)	668
	GC content	43.90%
**Annotation (database)**	NCBI nr	36,160 (47.60%)
	Swiss-Prot	25,057 (32.98%)
	Teleost Ensembl	31,372 (41.30%)
	KOG	19,515 (25.68%)
	KEGG	10,182 (13.40%)
	GO	26,263 (34.57%)

### Transcriptome annotation

**Fig 1:**
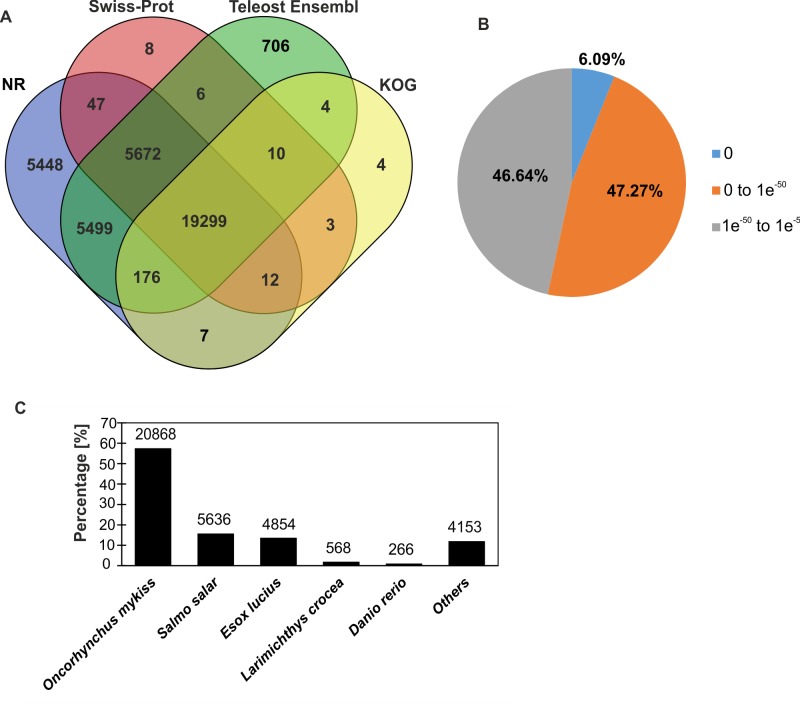
Characteristics of the homology search of sea trout transcripts. (A) Venn diagram illustrating the distribution of matches against four public databases (NCBI nr, Swiss-Prot, Teleost Ensembl and KOG databases). In total, 36,902 (48.57%) transcripts were annotated. (B) E-value and (C) species distribution of the best hits based on the NCBI nr database.

All the assembled 75,970 contigs were annotated by BLASTX searches against four protein databases (Table 1). In total, 36,902 (48.57%) contigs had hits in public databases, and 19,299 (52.30%) had hits in all four databases (Fig 1A). The E-value distribution of the top hits in the NCBI nr database indicated that 6.09% contigs showed excellent matches (E-value = 0) and that 47.27% had highly and moderately significant homology (≤1e-50, Fig 1B). Most of the matched contigs had hits in the salmonid family: rainbow trout (*O*. *mykiss*, 57.27%) and Atlantic salmon (*S*. *salar*, 15.47%), as expected. Moreover, 27.01% exhibited similarity to other organisms, mainly eukaryotes: fishes, mammals and birds (Fig 1C).

**Fig 2:**
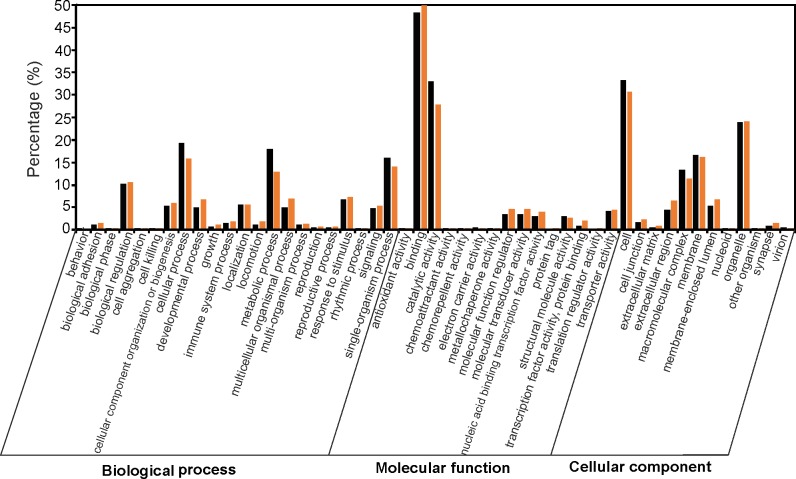
Gene Ontology comparative classification of the sea trout skin transcriptome (black) and multi-tissue transcriptome from the River Hayle and River Teign in southwest England (orange).

Based on the NCBI nr database best hits imported into Blast2Go, a total of 164,515 GO terms were assigned to 26,263 contigs, and on average, one contig was assigned to six GO terms. Of these, the biological process category was the most prevalent (89,576, 54.45%), followed by the cellular component category (40,896, 24.86%) and the molecular function category (34,043, 20.69%). Within the biological process category, contigs involved in cellular process (GO: 0009987) and metabolic process (GO: 0008152) were highly represented. In the cellular component category, cell (GO: 0005623) and organelle (GO: 0043226) were the most represented subcategories. Binding (GO: 0005488) and catalytic activity (GO: 0003824) were dominant groups within the molecular function category (Fig 2).

For functional prediction and classification, all assembled contigs were compared with the KOG database. In total, 19,515 (25.68%) contigs were classified into 24 functional categories. The seven largest categories were follows: general function prediction only (3,600, 16.28%), followed by signal transduction mechanisms (3,038, 13.74%); posttranslational modification, protein turnover, and chaperones (2,186, 9.89%); transcription (1,515, 6.85%); function unknown (1,396, 6.31%); cytoskeleton (1,380, 6.24%) and intracellular trafficking, secretion, and vesicular transport (1,353, 6.12%; Table 2).

**Table 2:** The eukaryotic orthologous groups (KOG) classification of the sea trout contigs.

KOG category	KOG functional class	Number of contigs
**Information storage and processing**		
	Translation, ribosomal structure and biogenesis [J]	871 (3.94%)
	RNA processing and modification [A]	941 (4.26%)
	Transcription [K]	1,515 (6.85%)
	Replication, recombination and repair [L]	376 (1.70%)
	Chromatin structure and dynamics [B]	390 (1.76%)
**Cellular processes and signaling**		
	Cell cycle control, cell division, chromosome partitioning [D]	667 (3.02%)
	Nuclear structure [Y]	120 (0.54%)
	Defense mechanismsm [V]	270 (1.22%)
	Signal transduction mechanisms [T]	3,038 (13.74%)
	Cell wall/membrane/envelope biogenesis [M]	136 (0.62%)
	Cell motility [N]	366 (1.66%)
	Cytoskeleton [Z]	1,380 (6.24%)
	Extracellular structures [W]	447 (2.02%)
	Intracellular trafficking, secretion, and vesicular transport [U]	1,353 (6.12%)
	Posttranslational modification, protein turnover, chaperones [O]	2,186 (9.89%)
**Metabolism**		
	Energy production and conversion [C]	595 (2.69%)
	Carbohydrate transport and metabolism [G]	547 (2.47%)
	Amino acid transport and metabolism [E]	457 (2.07%)
	Nucleotide transport and metabolism [F]	258 (1.17%)
	Coenzyme transport and metabolism [H]	129 (0.58%)
	Lipid transport and metabolism [I]	581 (2.63%)
	Inorganic ion transport and metabolism [P]	317 (1.43%)
	Secondary metabolites biosynthesis, transport and catabolism [Q]	172 (0.78%)
**P.ch.**		
	General function prediction only [R]	3,600 (16.28%)
	Function unknown [S]	1,396 (6.31%)

P.ch.–poorly characterized.

Pathway-based analysis revealed that 10,182 (13.40%) of the contigs were assigned to 344 pathways in the KEGG database. The five highly represented pathways include cancer (724), PI3K-Akt signaling (646), focal adhesion (568), endocytosis (543), proteoglycans in cancer (485), MAPK signaling (458) and regulation of actin cytoskeleton (451; S1 Table). Within the top five KO categories including metabolism, genetic information processing, environmental information processing, cellular process and organismal system, the subcategory signal transduction (6,197) was the most highly represented, followed by immune system (2,347), nervous system (1,542), transport and catabolism (1,320), translation (1,222), cellular community (1,197), and folding, sorting and degradation (1,196; Table 3).

**Table 3:** KEGG analysis of the sea trout skin transcriptome.

KO category	KO subcategory	Number of contigs
**Metabolism**		
	Overview	691 (2.60%)
	Carbohydrate metabolism	1,061 (3.99%)
	Energy metabolism	510 (1.92%)
	Lipid metabolism	750 (2.82%)
	Nucleotide metabolism	446 (1.68%)
	Amino acid metabolism	668 (2.51%)
	Metabolism of other amino acids	226 (0.85%)
	Glycan biosynthesis and metabolism	366 (1.37%)
	Metabolism of cofactors and vitamins	267 (1.00%)
	Metabolism of terpenoids and polyketides	52 (0.20%)
	Biosynthesis of other secondary metabolites	77 (0.29%)
	Xenobiotics biodegradation and metabolism	197 (0.74%)
**GIP**		
	Transcription	522 (1.96%)
	Translation	1,222 (4.59%)
	Folding, sorting and degradation	1,196 (4.49%)
	Replication and repair	319 (1.20%)
**EIP**		
	Membrane transport	50 (0.19%)
	Signaling molecules and interaction	584 (2.19%)
	Signal transduction	6,197 (23.28%)
**Cellular process**		
	Transport and catabolism	1,320 (4.96%)
	Cell motility	451 (1.69%)
	Cell growth and death	1,034 (3.88%)
	Cellular commiunity	1,197 (4.50%)
**Organismal system**		
	Immune system	2,347 (8.82%)
	Endocrine system	354 (1.33%)
	Circulatory system	715 (2.69%)
	Digestive system	760 (2.86%)
	Excretory system	354 (1.33%)
	Nervous system	1,542 (5.79%)
	Sensory system	344 (1.29%)
	Development	472 (1.77%)
	Environmental adaptation	328 (1.23%)

GIP–genetic information processing

EIP–environmental information processing.

Given the existence of contigs that were not annotated to known proteins in the four public databases by BLAST searches, we attempted to identify them by searching against the Rfam database [53]. As shown in S2 Table, a total of 526 transcripts without significant blast hits were identified as putative ncRNAs. UnaL2 LINE 3' element (UnaL2, 68.08%), ribosomal RNA (rRNA, 8.17%) and transfer RNA (tRNA, 7.22%) were the most abundant ncRNA families (S2 Table).

We compared the trout skin transcriptome to the multi-tissue transcriptome using BLASTn. While 68,697 (90.43%) contigs of the skin transcriptome were homologous with one of the 202,994 multi-tissue transcripts, 9.57% did not show any similarity and thus represent new sequence information. Of these 7,273 novel genes, 1,429 (19.65%) were annotated in at least one of the searched database. Additionally, 58,700 (85%) of homologous contigs have high similarity (>95%). We also investigated nucleotide-level comparisons by mapping skin transcriptome reads to the multi-tissue transcriptome. A total of 52,522 (69.14%) sequences were mapped to the reference transcriptome; of these sequences, 2.51% mapped perfectly and unambiguously. Moreover, GO annotation results were compared between these trout transcriptomes (Fig 2). In general, the main GO category assignment was similar: the biological process category was the most highly represented, followed by cellular component and molecular function categories. However, there were some minor differences in the distribution of the genes in these three categories. The most noticeable differences were observed in the biological process category. In this category the highest numbers of transcripts were associated with cellular process (19.11%), metabolic process (17.76%) and single-organismal process (15.77%) in the skin transcriptome and with cellular process (15.74%), single-organismal process (14.03%) and metabolic process (12.83%) in the multi-tissue transcriptome. Fisher’s exact test was applied to discover significantly over-represented GO terms in skin transcriptome. GO enrichment analysis revealed that the highest enrichment was associated with the molecular function category (51.28%), followed by the biological process category (32.48%) and cellular component category (16.24%; S3 Table). The over-represented biological process categories were metabolic process (e.g. methylation, GO:0032259, p-value = 1.9×10^−7^), cellular component and biogenesis (e.g. ribosome biogenesis, GO:0042254; p-value = 2.4×10^−5^), cellular process (e.g. cellular metabolic compound salvage, GO:0043094, p-value = 4.6×10^−9^) and single organismal process (e.g. oxidation reduction process, GO:0055114, p-value = 3×10^−11^). In molecular function category, over-represented were terms associated with catalytic activity (e.g. oxidoreductase activity, GO:0016491.p-value = 1×10^−27^; hydrolase activity, GO:0016787, p-value = 1.6×10^−6^), molecular transducer activity (e.g. scavenger receptor activity, GO:0005044;P-Value = 2×10^−7^), structural molecule activity (e.g. structural constituent of ribosome GO:0003735, p-value = 1.2×10^−17^) and binding (e.g. heterocyclic compound binding GO:1901363, p-value = 2.9×10^−8^; S3 Table).

### Mucins

The BLASTX results identified numerous contigs as showing significant homology to fish mucin genes. Searches in the created sea trout database identified 14 contigs that exhibited homology to mucins: 11 contigs showed homology to the SGFM family (Muc2, Muc5AC, Muc5B, I-Muc), and 3 showed homology to membrane-bound mucins (Muc12, Muc15, Muc17) (Table 4). Additionally, 14 contigs were obtained from the multi-tissue database and were annotated as Muc2, Muc5AC, Muc5B, I-Muc, Muc13 and Muc17 (S4 Table). Generally, the top hits of the BLASTX results were Atlantic salmon mucins, whereas of the top hits for ST_7108 (Muc15), ST_2730 were: northern pike (*Esox lucius*), brichardi cichlid (*Neolamprologus brichardi*), respectively. Furthermore, all contigs showed homology to *Salmo salar* genome (Table 4 and S4 Table).

**Table 4:** Classification of the mucins identified in skin transcriptome, according to the BLASTX and BLASTN searches.

Contig	Size (nt)	Accesion number	Annotation (NR)	Accession number (NR)	E-value	*Salmo salar* genome (ID)
ST_8618	1010	KY328730	*S*.*salar*, MUC2	XP_014025397	0	NC_027322.1
ST_3412	3340	KY328734	*S*.*salar*, MUC2	XP_013980029	0	NC_027309.1
ST_51843	1044	KY328735	*S*.*salar*, MUC2	XP_014039217	0	NW_012348149.1
ST_21871	842	KY328733	*S*.*salar*, MUC5B	XP_014031349	8e-55	NC_027325.1
ST_73144	444	KY328727	*S*.*salar*, MUC5AC	XP_014057394	3E-46	NC_027304.1
ST_75252	564	KY328731	*S*.*salar*, MUC5AC	XP_014041617	5E-116	NW_012353588.1
ST_36280	686	KY328732	*S*.*salar*, MUC5AC	XP_013982550	7E-155	NC_027301.1
ST_271	2877	KY328736	*S*.*salar*, MUC5B	XP_014031349	0	NC_027325.1
ST_4345	672	KY328737	*S*.*salar*, I-MUC	XP_014044558	2E-80	NC_027325.1
ST_39	2918	KY328738	*S*.*salar*, I-MUC	XP_014041914	0	NC_027309.1
ST_52675	413	KY328728	*S*.*salar*, I-MUC	XP_014044237	2E-77	NW_012347256.1
ST_35504	507	KY328729	*S*.*salar*, MUC12	XP_014037729	2E-43	NW_012343960.1
ST_7108	515	KY328739	*E*.*lucius*, MUC15	XP_010895353	8E-43	NC_027310.1
ST_2730	3528	KY328740	*N*.*brichardi*, MUC17	XP_006804478	2E-17	NC_027310.1

**Fig 3:**
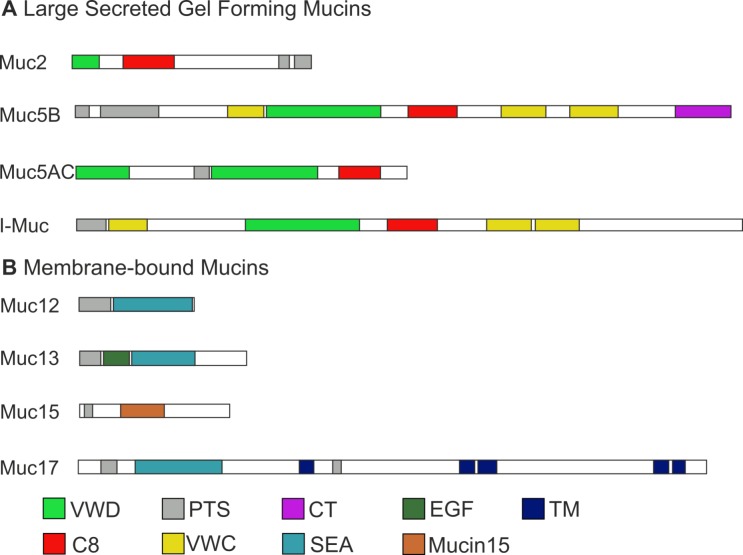
Schematic representation of the molecular structure of the eight sea trout skin mucins. Based on ST_51843, ST_271, ST_5641, ST_39, ST_35504, ST_27547, ST_7108, and ST_28361.

The SMART analysis revealed PTS domains in all annotated sequences typical for all mucins. As depicted in Fig 3, the sequences annotated as I-Muc, Muc2, Muc5AC, and Muc5B share VWD and C8 domain, which are typical for the SGFM subfamily. S2 Fig shows the deduced amino acid sequence of I-Muc together with sequence and domain alignments with orthologs from other species. A von Willebrand factor type C (VWC) domain was found in the sequences recognized as Muc5B and I-Muc, and a C-terminal cysteine knot-like domain (CT) was found in the sequences recognized as Muc5B. The sequences recognized as Muc12, Muc13 and Muc17 shared an extracellular proteolytic cleavage site (SEA domain) characteristic of membrane-bound mucins. The Muc13 sequences contained an EGF domain (before the SEA domain), and Muc17 sequences contained a TM domain. The sequence recognized as Muc15 possessed a Muc15 domain (Fig 3).

**Fig 4:**
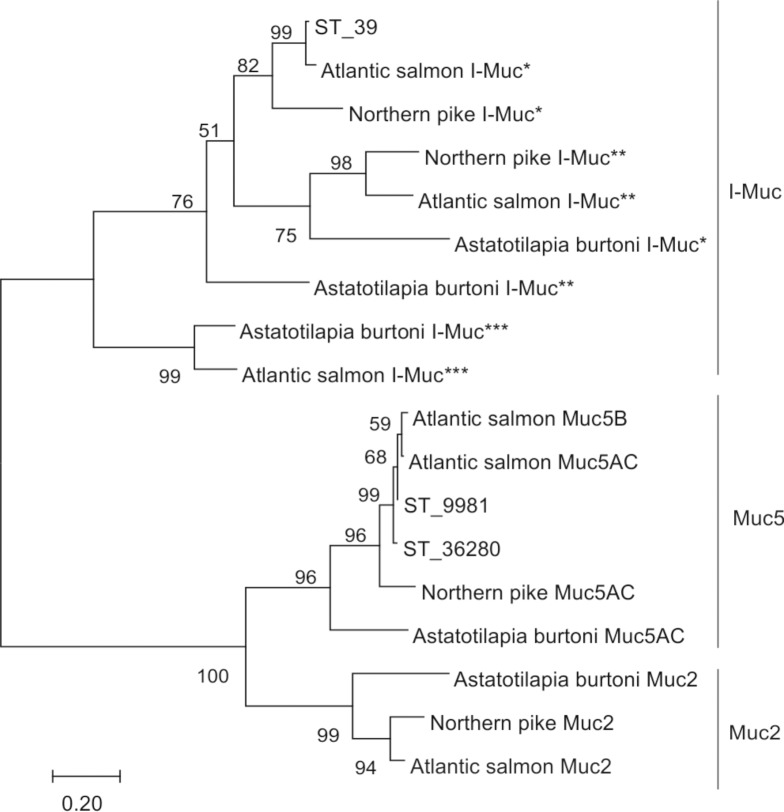
Unrooted phylogenetic tree showing the evolutionary relationship of the secreted gel-forming mucins (Muc2, Muc5AC, Muc5B, and I-Muc) in teleosts. The tree was constructed using multiple alignment of the VWD and C8 domains of the translated (ST_9981, ST_36280, ST_39) and selected teleost families: Salmonidae, Esocidae, and Cichlidae. The maximum likelihood phylogeny in MEGA 7 [61] was selected. The tree was bootstrapped 1,000 times. Accession numbers: Atlantic salmon I-Muc* (XP_014041914), northern pike I-Muc* (XP_012994242.1), northern pike I-Muc** (XP_012993966.1), Atlantic salmon I-Muc** (XP_013982567.1), *Astatotilapia burtoni* I-Muc* (XP_005941718.1), *A*. *burtoni* I-Muc** (XP_005946303.1); *A*. *burtoni* I-Muc*** (XP_005952623.1), Atlantic salmon I-Muc*** (XP_014038548.1), Atlantic salmon Muc5B (XP_014031349.1), Atlantic salmon Muc5AC (XP_014036802.1), northern pike Muc5AC (XP_010867519.2), *A*. *burtoni* Muc5AC (XP_005946314.1), *A*. *burtoni* Muc2 (XP_005952624.2), northern pike Muc2 (XP_012994223.1), Atlantic salmon Muc2 (XP_014040158.1).

The phylogenetic analysis of the mucins of salmonidae and other teleost fish revealed three major clades (Muc5, Muc2 and I-Muc) (Fig 4 and S3 Fig). Of note, the I-Muc genes are classified as a SGFM group with Muc2 and Muc5, in contrast to previous studies [39]. Contigs annotated as Muc5AC and Muc5B are part of the Muc5 protein family, although it cannot be definitely concluded whether they are type 5AC or 5B.

### Mucin biosynthesis

**Fig 5:**
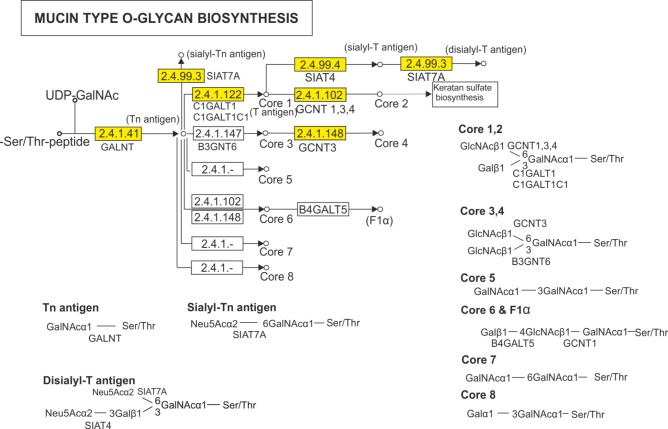
Map of the mucin type O-glycan biosynthesis pathway. Genes identified from the sea trout skin transcriptome are shown in yellow.

A total of 40 contigs from the sea trout skin transcriptome were assigned to the 7 enzymes in the mucin type O-glycan biosynthesis KEGG pathway (Fig 5). Of these 40 contigs, 29 contigs were mapped to GALNT enzymes (2.4.1.41), which includes 11 members of the N-acetylgalactosaminyltransferase family (GALNT 3, 4, 5, 6, 7, 8, 12, 14, 15 and 18). Moreover, 6 contigs were assigned to glycoprotein-N-acetylgalactosamine 3-beta-galactosyltransferases (2.4.1.122), and 2 contigs were mapped to glucosaminyl (N-acetyl) transferases (GCNT 3 and 4; 2.4.1.102/148). Furthermore, 3 contigs were annotated as sialyltransferases: SIAT4A (ST3GAL1), SIAT4B (ST3GAL2)–(2.4.99.3) and SIAT7 (ST6GALNAC2)–(2.4.99.4). In total, 140 genes that are implicated in synthesis, intracellular transport, and postsecretory modifications of mucins were identified in the sea trout skin transcriptome (S5 Table). In this study, 5 members of the protein disulfide isomerase (PDI) family and 45 members of the glycosyltransferase family were identified. Furthermore, transcripts of SAM-pointed domain-containing ETS transcription factor (SPDEF) and epidermal growth factor receptor (EGFR) were recognized. Two functional groups of genes encoding proteins associated with intracellular transport, RAB and SNARE (36 and 18 members respectively), were identified. Three functional groups of genes encoding proteins associated with postsecretory modifications of mucus gels were also identified. These proteins included 2 galectins, 4 aquaporins, and 13 ion channels, ion pumps and transporters. Six members of the C2 domain containing protein kinases family (PKC) and myristoylated alanine rich protein kinase C substrate (MARCKS) were also recognized.

### Immune pathways

Although brown trout multi-tissue transcriptomes have been previously characterized using RNA-seq [45], the immune response genes and pathways in the sea trout skin have not been characterized. GO classification identified 456 genes related to the immune response (GO: 0045087). Moreover, KEGG annotation identified 310 genes related to the immune system: these genes were distributed in 15 pathways. The dominant pathways included chemokine signaling (271), leukocyte transendothelial migration (264) and Fc gamma R-mediated phagocytosis (204) (Table 5). The highest number of retrieved genes in each pathway were found in the B cell receptor signaling pathway (74.55%), Fc gamma R-mediated phagocytosis (72.41%) and leukocyte transendothelial migration (72%) (Table 5). Combining annotations from GO, KEGG and BLAST searches revealed 954 genes potentially involved in immune reactions in the sea trout skin transcriptome (S6 Table).

**Table 5:** List of the immune pathways identified in the sea trout skin transcriptome.

Pathway name	KO identifier	Associated sequences	Mapped genes	Known genes	Identified/known genes (%)
Chemokine signaling pathway	ko04062	281	74	146	50.68%
Leukocyte transendothelial migration	ko04670	264	54	75	72.00%
Fc gamma R-mediated phagocytosis	ko04666	204	42	58	72.41%
T cell receptor signaling pathway	ko04660	199	50	84	59.52%
Toll-like receptor signaling pathway	ko04620	165	40	76	52.63%
B cell receptor signaling pathway	ko04662	149	41	55	74.55%
NOD-like receptor signaling pathway	ko04621	123	27	51	52.94%
Antigen processing and presentation	ko04612	121	22	41	53.66%
Natural killer cell mediated cytotoxicity	ko04650	115	35	85	41.18%
Fc epsilon RI signaling pathway	ko04664	108	29	45	64.44%
RIG-I-like receptor signaling pathway	ko04622	97	29	53	54.72%
Cytosolic DNA-sensing pathway	ko04623	77	30	51	58.82%
Complement and coagulation cascades	ko04610	71	38	69	55.07%
Hematopoietic cell lineage	ko04640	37	21	77	27.27%
Intestinal immune network for IgA production	ko04672	23	11	37	29.73%

### SSR detection

In total, 1,119 potential SSRs with an average length of 64.28 bp were identified in 3,134 (4.13%) contigs, and 130 of these contigs contained more than one SSR (Table 6). Among the identified repetitive elements, the most highly represented were di-nucleotide repeats, and AC/GT (703; 62.82%) was the dominating motif. Among the tri-nucleotide and tetra-nucleotide repeats, ACT/AGT and ACAT/ATGT (26 and 30, respectively) accounted for the most frequent motifs (S7 Table).

**Table 6:** Summary of SSRs identified from the sea trout skin transcriptome.

Searching item	Numbers
Total number of examined contigs	75,970
Total number of identified SSRs	1,119
Number of contigs containing SSR	3,134
Number of contigs containing more than 1 SSR	130
Di-nucleotide	990
Tri-nucleotide	48
Tretra-nucleotide	76
Penta-nucletoide	5

## Discussion

The innate immune system is the only known defense weapon of invertebrates and a fundamental defense mechanism of fish [65]. Skin and the mucosal layer form the first barrier against infection [29, 66]. The defensive role of mucus is well known and has been studied in several fish species [21, 26 and 28]. In the present study, we characterized the skin transcriptome of the sea trout using the Roche GS-FLX 454 pyrosequencing system, in order to identify putative immunogens, including mucins. We obtained over 479 Mb of raw sequence data and were able to assemble these reads into 75,970 contigs, 36,902 (48.57%) of which were annotated by BLAST searches against four public databases (Table 1), which is consistent with previous studies [45]. The high number of contigs (98%) were annotated by using the NCBI nr database, which suggest that this database is very reliable for annotation of non-model species. On the other hand this database exhibit high level of misannotation [67], so to ameliorate this problem other conservative and verified databases (Swiss-Prot, Ensembl and KOG) were used to confirm annotation. Moreover, these databases provide additional sequence information for 741 contigs e.g. RAB38b (putatively involved in mucin synthesis), vesicle transport through interaction with t-SNAREs 1A (VTI1A, involved in vesicular transport) [68], peroxiredoxin (antioxidant). These results provide a valuable resource for further brown trout genome studies. Furthermore, most of the species on the top hits list of annotated genes were fishes, suggesting the high quality of the assembled transcripts and high levels of sequence conservation and homology with other fish species, mainly salmonids (Fig 1C). A large number of contigs were assigned to GO terms in three main categories, including biological process, molecular function and cellular component (Fig 2). GO analysis of the trout skin was compared with a transcriptome from a previous study [45]. The proportion of annotated contigs and GO terms were comparable, despite the different number of sequences used to retrieve GO terms (202,994 versus 75,970) and the different sequencing system (Illumina versus 454). This result suggests that the dataset from this study includes a wide variety and full view of the diverse structural, regulatory and metabolic proteins expressed in the sea trout skin. According to the GO enrichment results, terms associated with biological processes (e.g. metabolic process) were enriched, indicating the basic functions of trout skin (S3 Table). Moreover, ribosomal proteins (40s and 60s) were over-expressed, similarly with the skin of channel catfish [69]. In the level two GO category of molecular function, enriched were terms involved in catalytic activity and molecular transducer activity, including oxidoreductase activity (e.g. superoxide dismutase), hydrolase activity (e.g. leukotriene A-4 hydrolase) and scavenger receptor activity (e.g. scavenger receptor class A). All these genes are involved in stress reaction and immune response against pathogens in Atlantic salmon and other teleost [29, 70, 71]. Furthermore, 7,273 contigs did not match any sequences identified in the multi-tissue transcriptome. These contigs may represent novel or specific genes for sea trout or for the skin transcriptome.

Genes identified in the KEGG database provide a resource for functional information and a better understanding of the physiology of organisms [72]. The annotated contigs represent a wide array of KEGG-linked pathways (Table 3 and S1 Table). The predominantly represented KO category was organismal system (7,216), with highly represented subcategories including immune system and nervous system pathways and environmental information processing (6,831), with signal transduction pathway being predominant (Table 3). The most highly represented pathways were associated with signal transduction and involved in the immune system, such as the PI3K-Akt signaling pathway (secretion of proinflammatory cytokines) [73], MAPK signaling pathway (production of immunomodulatory cytokines) [74] and Rap1 signaling pathway (essential for changes in B cell morphology) [75]. Other highly represented pathways included those involved in various cellular functions, including cell proliferation, differentiation, motility, and regulation of gene expression, suggesting high cellular process activity in the trout skin, which is comparable with previous studies of fish skin and which suggest the importance of this pathway in fish skin [28]. Moreover, many genes associated with the immune system were expressed in the trout skin. The identified immunogens represent between 51% to 75% of the known genes in each pathway, except natural killer cell-mediated cytotoxicity, hematopoietic cell lineage and intestinal immune network for IgA production (Table 5). The highest numbers of sequences were assigned to chemokine signaling and leukocyte transendothelial migration pathways, which are both differentially activated in other fish (*Pseudosciaena crocea*) infected with *Aeromonas hydrophila* [76]. These results seem to be consistent with previous skin studies of mud loach [28], with two exceptions. In sea trout, more genes were restored in the complement and coagulation cascades (55% versus 31% in mud loach), and in contrast, antigen processing and presentation was less represented (54% versus 75%). Both GO and KEGG analyses revealed significantly enriched GO terms and pathways associated with the immune system (S6 Table). In addition to KEGG pathways, our high-throughput sequencing effort revealed the presence of numerous (954) molecules involved in the immune response: cytokines, antimicrobial peptides (AMPs), lectins, galectins, heat shock proteins, granzymes, immunoglobulins, complement components and cathepsins.

Antimicrobial peptides are oligopeptides with a varying number (from five to over a hundred) of amino acids [77], playing crucial role in innate immune defences against pathogen. They display a broad spectrum of activity against numerous pathogenic organisms including Gram-positive and Gram-negative bacteria, yeast, fungi, and parasites with little or no toxicity to host cells [25]. AMPs activity have been tested against few bacterial pathogens [78, 79]. In present study, we identified several contigs presenting homology to cathelicidins, β-defensin, hepcidin and H2A.

The complement component system is a cascade of a large number of distinct plasma proteins that react with one another to opsonize pathogens and induce a series of inflammatory responses [80] essential in innate immune system. In vertebrates, the three pathways of activation (classical, alternative and lectin pathway) lead to generate C3-convertase, which activated the central component C3 [81]. We have identified several sequences presenting homology to C1, C3, C4, C5, C6, C7, and C8. Only the C2 and C9 (component seems not to be represented. Moreover, we identified receptors and other proteins involved in the complement component cascade, including the complement receptor type 1and type 2 (CR1, CR2), the complement factor H, D.

Immunoglobulins are Y-shaped glycoproteins produced mainly by plasma cells and play key roles in the mucosal adaptive system, specifically recognizing and binding to particular antigens, such as bacteria [25]. There are three major types of immunoglobulins in teleost fish: IgM, IgD and IgT/IgZ, the latter of which is unique for teleost fish [82, 83]. In present study, we identified several contigs presenting similarity to IGHD and IGHM.

Among other immune-related proteins, several proteins were identified as being related to immune mechanisms in Atlantic salmon and rainbow trout infected by *A*. *salmonicida*. These proteins include ferritin, voltage-dependent anion channel 2 (VDAC2), GTPase IMAP family member 7 (GiMAP7), protease subunit alpha type (PSMA1), annexin A11 (ANXA11), lysozyme, and alkaline phosphatase (AKP) [28, 29] (S6 Table), which were not previously reported for the sea trout transcriptome.

Mucins are high molecular weight glycoproteins and are the main constituents of fish skin mucus, which can produce a protective gel to bind a range of bacteria and which constitutes an important part of the mucosal defense against infection [37]. The mucins and other genes involved in mucus synthesis remain poorly characterized. In this study, 7 types of mucins (14 contigs) were identified (Table 4). The molecular identity of these mucins was established on the basis of BLAST annotations, domain searches and phylogenetic analysis. All annotated mucins contain a PTS domain (Fig 3). The number of repeats rich in proline, threonine and serine and the amino acid sequence depend on the mucin type [30]. In contigs annotated as intestinal mucin, a VWD domain was found, indicating a relationship to the SGFM mucin family (Fig 3 and S3 Fig), although this domain can also be found in other non-mucin proteins [84]. However, the sea trout sequences found in the present study also share cysteine-rich domains with other bony fishes (Fig 3 and S3 Fig). The phylogenetic analysis suggested that the VWD and C8 domains have been conserved throughout the evolution of vertebrates, consistent with previous studies [39, 85]. It is noteworthy that I-Muc was categorized as a SGFM based on phylogenetic analysis and domain characteristics which is in contrast to the results of previous studies [39] (Figs 3 and 4). Moreover, phylogenetic analysis of intestinal mucins in a representative teleost group suggested that I-Muc family might consist of three subfamilies (S3 Fig). Notably, the identified mucins are the first reported trout sequences in the GenBank database. Furthermore, we reported the first protein sequence identified as Muc15 for the Salmonidae family. The presence of characteristic domains such as VWD, C8, SEA and TM strongly supports conclusion that the sea trout mucins are real mucin genes, instead of genes that only reassemble mucins (Muc-like genes). Mucins are characterized by their extensive O-glycosylation [86]. The biosynthesis of O-glycans starts from the transfer of N-acetylgalactosamine (GalNAc) to serine or threonine [87]. The first GalNAc may be extended with sugars such as galactose, N-acetylglucosamine, fucose, or sialic acid, but not mannose, glucose, or xylose. Depending on the sugars added, there are four common O-glycan core structures, cores 1 through 4, and an additional four, cores 5 through 8 [87]. In this study, 7 enzymes assigned to the mucin type O-glycan biosynthesis KEGG pathway were identified (Fig 5), which is similar to the findings of a previous study [28], suggesting that only cores 1 through 4 are activated in fish. Moreover, 11 members of the polypeptide N-acetyl-galactosaminyltransferase family (EC2.4.1.41) were expressed in the skin of sea trout. These enzymes belong to a large enzyme family (with at least 15 members) with tissue-specific expression and different substrate specifies [88]; these enzymes are active at the first step of glycosylation. Furthermore, 129 other genes associated with mucin synthesis were identified (S5 Table). The results of this study provide a valuable resource for characterizing mucus secretion in sea trout.

Transcriptome- and EST-based markers are important resource for determining functional genetic variation [89]. Moreover, because transcriptome-based SSRs mainly occur in the protein-coding regions of annotated genes, they are better at identifying phenotypic associations [90]. Among the various molecular markers, SSRs are highly polymorphic, are relatively easy to develop and serve as a rich source of diversity [91]. In this study, we identified 1,119 potential SSRs with predominant di-nucleotide repeats (Table 6).

## Conclusions

This study investigated the skin transcriptome of the sea trout using the Roche GS-FLX 454 pyrosequencing system. We generated 75,970 contigs, 48.57% of which were annotated by BLAST searches. A large number of contigs were classified according to GO and KO terms, which represent multiple signaling pathways and processes. Many of these terms are related to the immune response including mucins and mucus secretion. To our knowledge, this study is the first to describe the skin transcriptome of sea trout.

## Supporting information

S1 Fig Sea trout skin transcriptome analysis pipeline. (TIF)

S2 Fig Multiple alignment of the amino acid sequences of sea trout I-Muc with other teleost. (TIF)

S3 Fig Unrooted phylogenetic tree showing the evolutionary relationship of the I-Muc in teleost. The tree was constructed using multiple alignment of the VWD and C8 domains of the translated (ST_10480 and ST_39) and selected teleost families: Salmonidae, Esocidae, Poeciliidae, Cyprinodontidae, and Cichlidae. The maximum likelihood phylogeny in MEGA 7 [61] was selected. The tree was bootstrapped 1000 times. Accession numbers: Atlantic salmon* (XP_014041914.1), northern pike* (XP_012994242.1), *A*. burtoni* (XP_005941718.1), cave molly* (XP_014867637.1), northern pike** (XP_012993966.1), Atlantic salmon** (XP_013982567.1), *A*.burtoni** (XP_005946303.1), sheepshead minnow* (XP_015252020.1), cave molly** (XP_014863832.1), sheepshead minnow** (XP_015259098.1), cave molly*** (XP_014832181.1), *A*. burtoni*** (XP_005952623.1), Atlantic salmon*** (XP_014038548.1).
(TIF)

S1 Table Summary of sequences involved in the KEGG pathways and KO categories. (PDF)

S2 Table Classification of identified non-coding RNAs. (PDF)

S3 Table Significantly overrepresented GO terms in the sea trout skin transcriptome (FDR<0.05). (PDF)

S4 Table Classification of the mucins identified in multi-tissue transcriptome, according to the BLASTX and BLASTN searches. (PDF)

S5 Table Genes involved in mucin biosynthesis and mucus production in sea trout skin transcriptome. (PDF)

S6 Table List of immunity-related genes found in sea trout skin transcriptome. (PDF)

S7 Table Distribution of EST-SSRs based on motif types. (PDF)
