# Supplementary figures and images for: De novo assembly of the sea trout (Salmo trutta m. trutta) skin transcriptome to identify putative genes involved in the immune response and epidermal mucus secretion
Source: PLoS One. 2017 Feb 17;12(2):e0172282. doi: 10.1371/journal.pone.0172282 (PMC5315281; doi:10.1371/journal.pone.0172282)

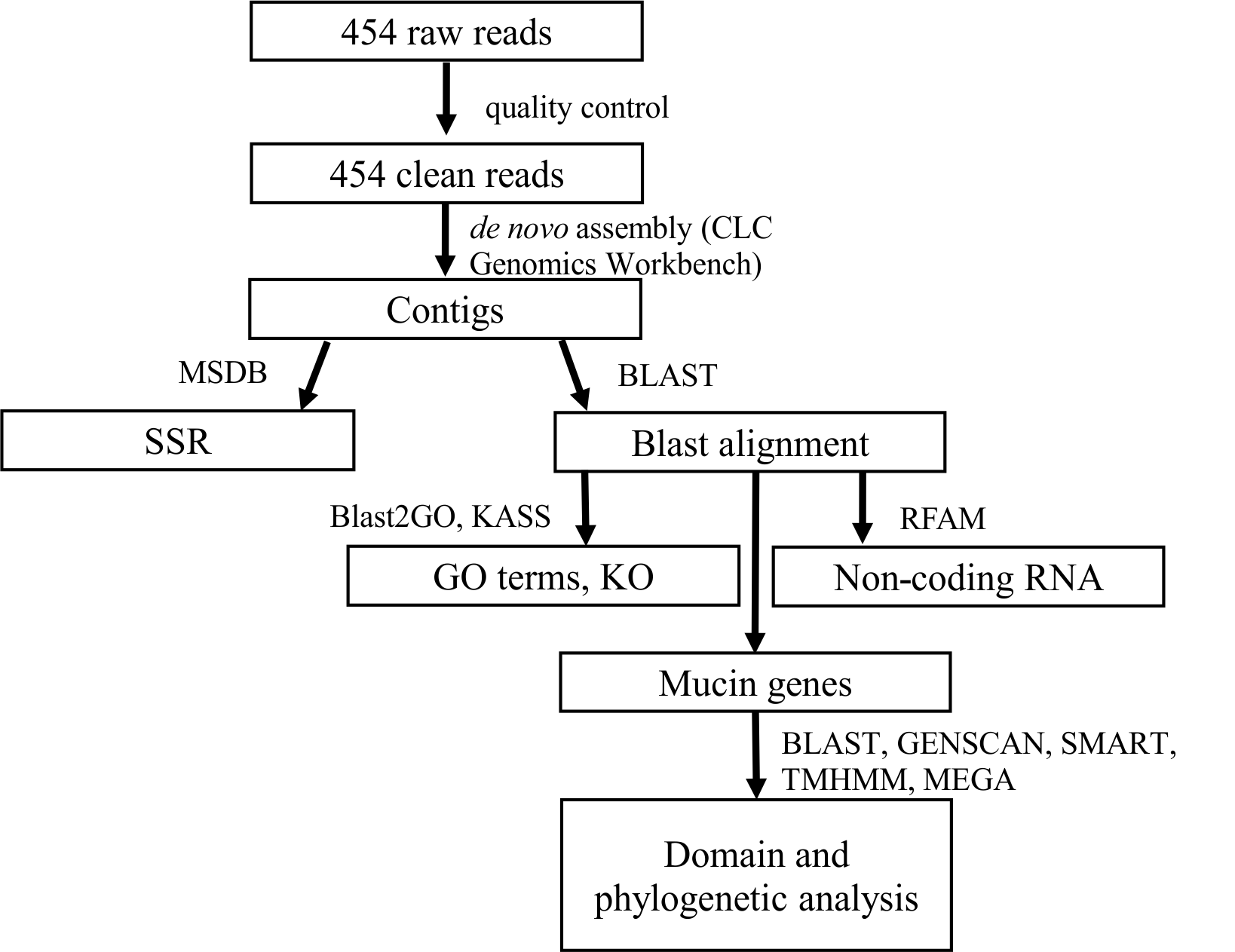

Supplement: S1 Fig — (TIF) [file pone.0172282.s001.tif]

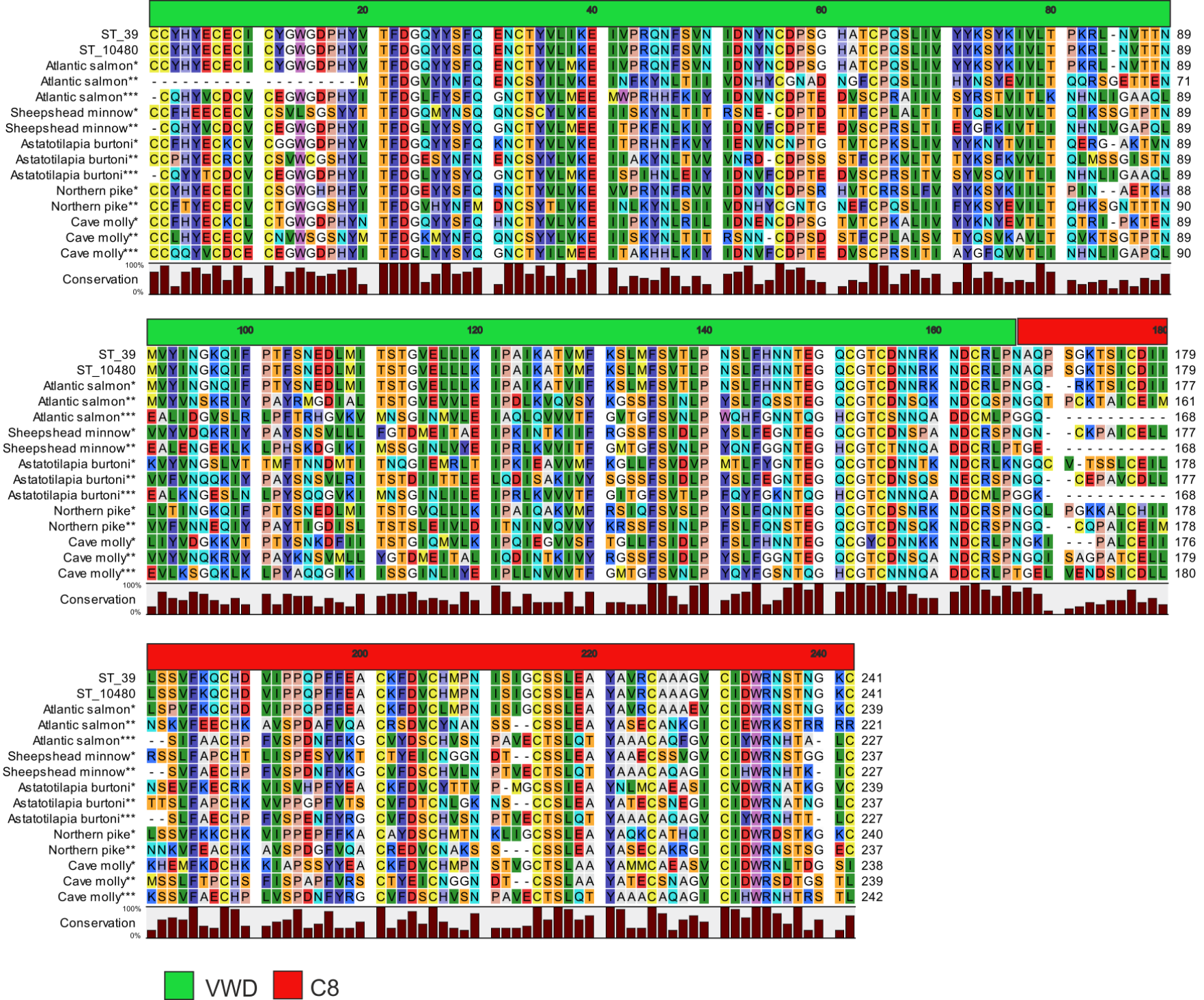

Supplement: S2 Fig — (TIF) [file pone.0172282.s002.tif]

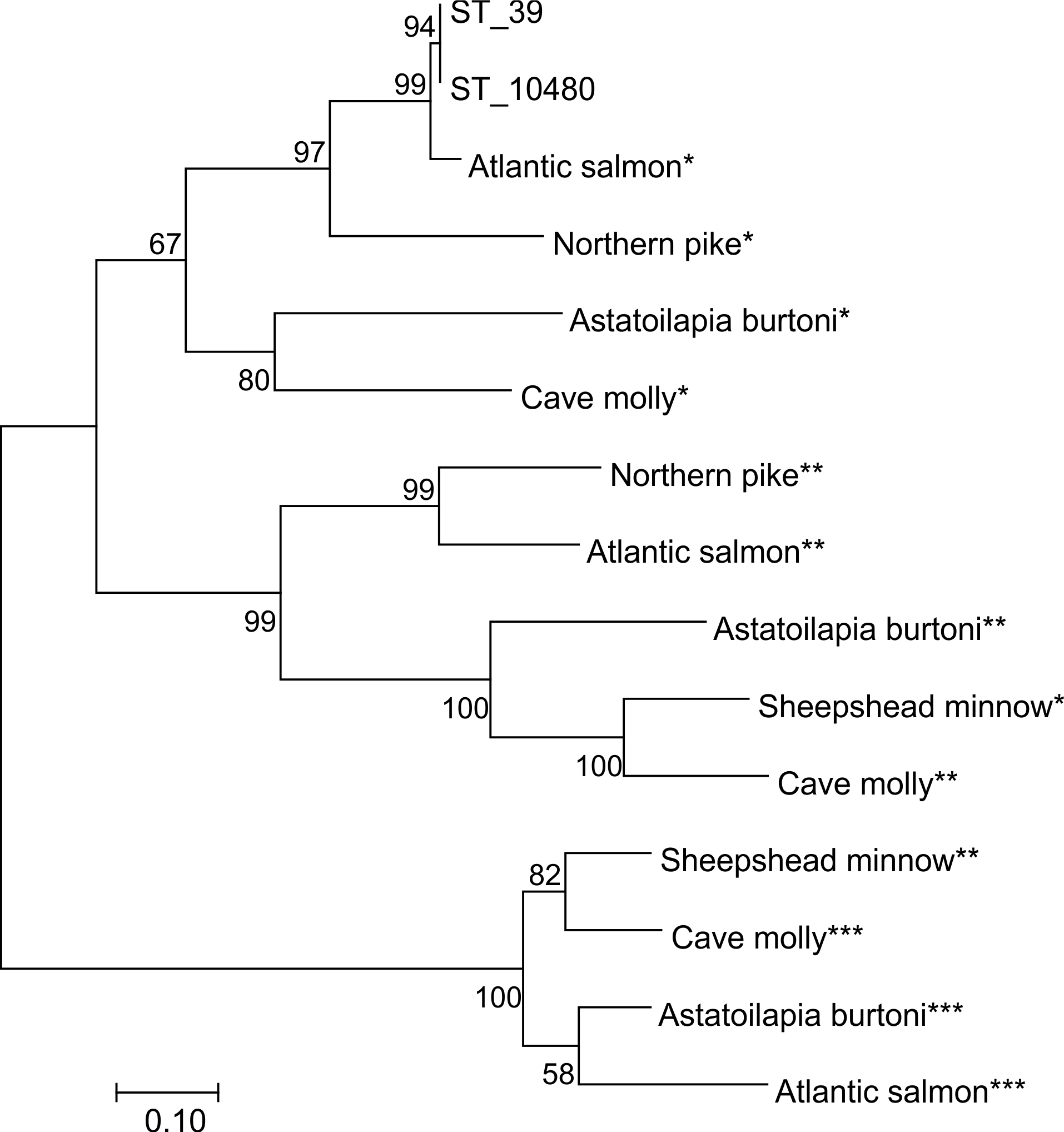

Supplement: S3 Fig — The tree was constructed using multiple alignment of the VWD and C8 domains of the translated (ST_10480 and ST_39) and selected teleost families: Salmonidae, Esocidae, Poeciliidae, Cyprinodontidae, and Cichlidae. The maximum likelihood phylogeny in MEGA 7 [61] was selected. The tree was bootstrapped 1000 times. Accession numbers: Atlantic salmon* (XP_014041914.1), northern pike* (XP_012994242.1), A. burtoni* (XP_005941718.1), cave molly* (XP_014867637.1), northern pike** (XP_012993966.1), Atlantic salmon** (XP_013982567.1), A.burtoni** (XP_005946303.1), sheepshead minnow* (XP_015252020.1), cave molly** (XP_014863832.1), sheepshead minnow** (XP_015259098.1), cave molly*** (XP_014832181.1), A. burtoni*** (XP_005952623.1), Atlantic salmon*** (XP_014038548.1). (TIF) [file pone.0172282.s003.tif]
