# Supplementary material for: De novo assembly of the sea trout (Salmo trutta m. trutta) skin transcriptome to identify putative genes involved in the immune response and epidermal mucus secretion
Source: PLoS One. 2017 Feb 17;12(2):e0172282. doi: 10.1371/journal.pone.0172282 (PMC5315281; doi:10.1371/journal.pone.0172282)
Supplement: S1 Table — (PDF) [file pone.0172282.s004.pdf]

S1 Table. Summary of sequences involved in the KEGG pathways and KO categories.

**Pathways**	**Number of sequences**
2-Oxocarboxylic acid metabolism	49
ABC transporters	47
Acute myeloid leukemia	133
Adherens junction	252
Adipocytokine signaling pathway	136
Adrenergic signaling in cardiomyocytes	339
Aflatoxin biosynthesis	6
African trypanosomiasis	40
Alanine, aspartate and glutamate metabolism	89
Alcoholism	162
Aldosterone synthesis and secretion	127
Aldosterone-regulated sodium reabsorption	58
Allograft rejection	25
alpha-Linolenic acid metabolism	20
Alzheimer's disease	396
Amino sugar and nucleotide sugar metabolism	96
Aminoacyl-tRNA biosynthesis	89
Aminobenzoate degradation	16
Amoebiasis	236
Amphetamine addiction	107
AMPK signaling pathway	307
Amyotrophic lateral sclerosis (ALS)	80
Antigen processing and presentation	121
Apoptosis	135
Arachidonic acid metabolism	70
Arginine and proline metabolism	105
Arginine biosynthesis	53
Arrhythmogenic right ventricular cardiomyopathy (ARVC)	191
Ascorbate and aldarate metabolism	20
Asthma	9
Autoimmune thyroid disease	26
Axon guidance	185
B cell receptor signaling pathway	149
Bacterial invasion of epithelial cells	229
Bacterial secretion system	3
Basal cell carcinoma	57
Basal transcription factors	65
Base excision repair	57
Benzoate degradation	8
beta-Alanine metabolism	57
Betalain biosynthesis	3
Bile secretion	74

Biosynthesis of amino acids	206
Biosynthesis of ansamycins	5
Biosynthesis of unsaturated fatty acids	39
Biosynthesis of vancomycin group antibiotics	1
Biotin metabolism	5
Bisphenol degradation	2
Bladder cancer	85
Butanoate metabolism	39
Butirosin and neomycin biosynthesis	10
C5-Branched dibasic acid metabolism	3
Caffeine metabolism	3
Calcium signaling pathway	251
cAMP signaling pathway	308
Caprolactam degradation	15
Carbapenem biosynthesis	1
Carbohydrate digestion and absorption	56
Carbon fixation in photosynthetic organisms	70
Carbon fixation pathways in prokaryotes	48
Carbon metabolism	321
Cardiac muscle contraction	188
Cell adhesion molecules (CAMs)	125
Cell cycle	245
Cell cycle - Caulobacter	10
Cell cycle - yeast	173
Central carbon metabolism in cancer	159
cGMP - PKG signaling pathway	298
Chagas disease American trypanosomiasis	213
Chemical carcinogenesis	57
Chemokine signaling pathway	281
Chloroalkane and chloroalkene degradation	7
Chlorocyclohexane and chlorobenzene degradation	1
Choline metabolism in cancer	192
Cholinergic synapse	156
Chronic myeloid leukemia	145
Circadian entrainment	157
Circadian rhythm	84
Circadian rhythm - fly	29
Circadian rhythm - plant	19
Citrate cycle (TCA cycle)	95
Cocaine addiction	73
Collecting duct acid secretion	42
Colorectal cancer	175
Complement and coagulation cascades	71
Cyanoamino acid metabolism	5
Cysteine and methionine metabolism	95
Cytokine-cytokine receptor interaction	123

Cytosolic DNA-sensing pathway	77
Degradation of aromatic compounds	6
D-Glutamine and D-glutamate metabolism	19
Dilated cardiomyopathy (DCM)	359
DNA replication	67
Dopaminergic synapse	245
Dorso-ventral axis formation	43
Drug metabolism - cytochrome P 450	43
Drug metabolism - other enzymes	47
ECM-receptor interaction	241
Endocrine and other factor-regulated calcium reabsorption	100
Endocytosis	543
Endometrial cancer	127
Epithelial cell signaling in Helicobacter pylori infection	183
Epstein-Barr virus infection	438
ErbB signaling pathway	159
Estrogen signaling pathway	213
Ether lipid metabolism	67
Fanconi anemia pathway	39
Fat digestion and absorption	39
Fatty acid biosynthesis	32
Fatty acid degradation	82
Fatty acid elongation	53
Fatty acid metabolism	109
Fc epsilon RI signaling pathway	108
Fc gamma R-mediated phagocytosis	204
Flavone and flavonol biosynthesis	2
Fluorobenzoate degradation	1
Focal adhesion	568
Folate biosynthesis	16
FoxO signaling pathway	283
Fructose and mannose metabolism	83
GABAergic synapse	109
Galactose metabolism	59
Gap junction	171
Gastric acid secretion	122
Geraniol degradation	5
Glioma	114
Glucagon signaling pathway	227
Glutamatergic synapse	140
Glutathione metabolism	87
Glycerolipid metabolism	82
Glycerophospholipid metabolism	122
Glycine, serine and threonine metabolism	82
Glycolysis / Gluconeogenesis	152
Glycosaminoglycan biosynthesis - chondroitin sulfate / dermatan sulfate	17

Glycosaminoglycan biosynthesis - heparan sulfate / heparin	11
Glycosaminoglycan biosynthesis - keratan sulfate	13
Glycosaminoglycan degradation	21
Glycosphingolipid biosynthesis - ganglio series	18
Glycosphingolipid biosynthesis - globo series	16
Glycosphingolipid biosynthesis - lacto and neolacto series	26
Glycosylphosphatidylinositol (GPI-anchor biosynthesis)	21
Glyoxylate and dicarboxylate metabolism	68
GnRH signaling pathway	194
Graft-versus-host disease	25
Hedgehog signaling pathway	56
Hematopoietic cell lineage	37
Hepatitis B	292
Hepatitis C	245
Herpes simplex infection	345
HIF- signaling pathway	213
Hippo signaling pathway	306
Hippo signaling pathway -fly	181
Histidine metabolism	20
Homologous recombination	29
HTLV-I infection	443
Huntington's disease	384
Hypertrophic cardiomyopathy (HCM)	360
Inflammatiory bowel disease (IBD)	67
Inflammatory mediator regulation of TRP channels	155
Influenza A	320
Inositol phosphate metabolism	101
Insulin resistance	231
Insulin secretion	111
Insulin signaling pathway	302
Intestinal immune network for IgA production	23
Isoquinoline alkaloid biosynthesis	14
Jak-STAT signaling pathway	141
Legionellosis	131
Leishmaniasis	144
Leukocyte transendothelial migration	264
Limonene and pinene degradation	3
Linoleic acid metabolism	21
Lipoic acid metabolism	4
Lipopolysaccharide biosynthesis	1
Long-term depression	117
Long-term potentiation	132
Lysine biosynthesis	3
Lysine degradation	104
Lysosome	267
Malaria	67

MAPK signaling pathway	458
MAPK signaling pathway - fly	36
MAPK signaling pathway - yeast	74
Maturity onset diabetes of the young	11
Measles	207
Meiosis - yeast	135
Melanogenesis	168
Melanoma	90
Metabolism of xenobiotics by cytochrome P 450	49
Methane metabolism	72
MicroRNAs in cancer	319
Mineral absorption	44
Mismatch repair	35
Monobactam biosynthesis	6
Morphine addiction	79
mRNA surveillance pathway	233
mTOR signaling pathway	140
Mucin type O-glycan biosynthesis	29
Naphthalene degradation	2
Natural killer cell mediated cytotoxicity	115
Neuroactive ligand-receptor interaction	95
Neurotrophin signaling pathway	275
NF-kappa B signaling pathway	150
N-Glycan biosynthesis	76
Nicotinate and nicotinamide metabolism	52
Nicotine addiction	5
Nitrogen metabolism	28
Nitrotoluene degradation	1
NOD-like receptor signaling pathway	123
Non-alcoholic fatty liver disease NAFLD	333
Non-homologous end-joining	19
Non-small cell lung cancer	99
Notch signaling pathway	83
Novobiocin biosynthesis	1
Nucleotide excision repair	73
Olfactory transduction	52
One carbon pool by folate	35
Oocyte meiosis	220
Osteoclast differentiation	244
Other glycan degradation	21
Other types of O-glycan biosynthesis	38
Ovarian Steroidogenesis	48
Oxidative phosphorylation	265
Oxytocin signaling pathway	356
p53 signaling pathway	116
Pancreatic cancer	157

Pancreatic secretion	137
Pantothenate and CoA biosynthesis	21
Parkinson's disease	278
Pathogenic Escherichia coli infection	178
Pathways in cancer	724
Pentose and glucuronate interconversions	34
Pentose phosphate pathway	74
Peroxisome	120
Pertussis	162
Phagosome	336
Phenylalanine metabolism	24
Phenylalanine, tyrosine and tryptophan biosynthesis	13
Phenylpropanoid biosynthesis	1
Phosphatidylinositol signaling system	140
Phospholipase D signaling pathway	218
Phosphonate and phosphinate metabolism	10
Photosynthesis	4
Phototransduction	23
Phototransduction - fly	81
PI3K-Akt signaling pathway	646
Plant-pathogen interaction	39
Platelet activation	313
Polyketide sugar unit biosynthesis	1
Porphyrin and chlorophyll metabolism	52
PPAR signaling pathway	98
Primary bile acid biosynthesis	18
Primary immunodeficiency	22
Prion diseases	64
Progesterone-mediated oocyte maturation	160
Prolactin signaling pathway	127
Propanoate metabolism	69
Prostate cancer	194
Proteasome	142
Protein digestion and absorption	162
Protein export	48
Protein processing in endoplasmic reticulum	433
Proteoglycans in cancer	485
Proximal tubule bicarbonate reclamation	51
Purine metabolism	267
Pyrimidine metabolism	179
Pyruvate metabolism	91
Rap signaling pathway	360
Ras signaling pathway	322
Regulation of actin cytoskeleton	451
Regulation of autophagy	54
Regulation of lipolysis in adipocyte	101

Renal cell carcinoma	155
Renin secretion	102
Renin-angiotensin system	29
Retinol metabolism	48
Retrograde endocannabinoid signaling	121
Rheumatoid arthritis	110
Riboflavin metabolism	6
Ribosome	324
Ribosome biogenesis in eukaryotes	169
RIG-I-like receptor signaling pathway	97
RNA degradation	180
RNA polymerase	54
RNA transport	407
Salivary secretion	112
Salmonella infection	233
Selenocompound metabolism	34
Serotonergic synapse	135
Sesquiterpenoid and triterpenoid biosynthesis	3
Shigellosis	187
Signaling pathways regulating pluripotency of stem cells	206
Small cell lung cancer	200
SNARE interactions in vesicular transport	47
Sphingolipid metabolism	71
Sphingolipid signaling pathway	269
Spliceosome	403
Staphylococcus aureus infection	37
Starch and sucrose metabolism	77
Steroid biosynthesis	24
Steroid hormone biosynthesis	32
Streptomycin biosynthesis	19
Styrene degradation	4
Sulfur metabolism	23
Sulfur relay system	12
Synaptic vesicle cycle	112
Synthesis and degradation of ketone bodies	17
Systemic lupus erythematosus	88
T cell receptor signaling pathway	199
Taste transduction	33
Taurine and hypotaurine metabolism	14
Terpenoid backbone biosynthesis	34
TGF-beta signaling pathway	178
Thiamine metabolism	4
Thyroid cancer	82
Thyroid hormone signaling pathway	245
Thyroid hormone synthesis	114
Tight junction	393

TNF signaling pathway	204
Toll-like receptor signaling pathway	165
Toluene degradation	1
Toxoplasmosis	257
Transcriptional misregulation in cancers	283
Tropane, piperidine and pyridine alkaloid biosynthesis	11
Tryptophan metabolism	51
Tuberculosis	332
Two-component system	29
Type I diabetes mellitus	39
Type II diabetes mellitus	77
Tyrosine metabolism	34
Ubiquinone and other terpenoid-quinone biosynthesis	16
Ubiquitin mediated proteolysis	334
Valine, leucine and isoleucine biosynthesis	5
Valine, leucine and isoleucine degradation	97
Various types of N-glycan biosynthesis	58
Vascular smooth muscle contraction	188
Vasopressin-regulated water reabsorption	103
VEGF signaling pathway	132
Vibrio cholerae infection	150
Viral carcinogenesis	431
Viral myocarditis	177
Vitamin B6 metabolism	8
Vitamin digestion and absorption	14
Wnt signaling pathway	255
