# Supplementary material for: De novo assembly of the sea trout (Salmo trutta m. trutta) skin transcriptome to identify putative genes involved in the immune response and epidermal mucus secretion
Source: PLoS One. 2017 Feb 17;12(2):e0172282. doi: 10.1371/journal.pone.0172282 (PMC5315281; doi:10.1371/journal.pone.0172282)
Supplement: S2 Table — (PDF) [file pone.0172282.s005.pdf]

S2 Table. Classification of identified non-coding RNAs.

**NcRNA family**	**Number of ncRNAs identified**	**Number of associated transcripts**
snoRNA	15	26
microRNA	10	18
snRNA	4	7
rRNA	3	43
Iron response element	2	5
miRNA	2	2
methionine adenosyltransferase	2	3
5.8S ribosomal RNA	1	3
5S ribosomal RNA	1	5
Antizyme RNA frameshifting stimulation element	1	4
Histone 3' UTR stem-loop	1	7
potassium channel RNA editing signal	1	5
UnaL2 LINE 3' element	1	358
U1A polyadenylation inhibition element (PIE)	1	2
tRNA	1	38
**Total**	46	526
