# Supplementary material for: De novo assembly of the sea trout (Salmo trutta m. trutta) skin transcriptome to identify putative genes involved in the immune response and epidermal mucus secretion
Source: PLoS One. 2017 Feb 17;12(2):e0172282. doi: 10.1371/journal.pone.0172282 (PMC5315281; doi:10.1371/journal.pone.0172282)
Supplement: S3 Table — (PDF) [file pone.0172282.s006.pdf]

S3 Table. Significantly overrepresented GO terms in the sea trout skin transcriptome (FDR<0.05).

GO-ID	Term	Category	FDR	P-Value
GO:0003824	catalytic activity	F	1.40E-39	5.08E-43
GO:0097159	organic cyclic compound binding	F	4.57E-05	1.39E-07
GO:1901363	heterocyclic compound binding	F	1.02E-05	2.86E-08
GO:0003676	nucleic acid binding	F	9.77E-09	1.92E-11
GO:0036094	small molecule binding	F	2.14E-03	9.54E-06
GO:1901265	nucleoside phosphate binding	F	4.21E-05	1.24E-07
GO:0000166	nucleotide binding	F	4.21E-05	1.24E-07
GO:0016787	hydrolase activity	F	4.52E-04	1.62E-06
GO:0016740	transferase activity	F	2.52E-02	1.44E-04
GO:0032549	ribonucleoside binding	F	3.86E-02	2.34E-04
GO:0001883	purine nucleoside binding	F	3.43E-02	2.02E-04
GO:0032550	purine ribonucleoside binding	F	3.56E-02	2.14E-04
GO:0035639	purine ribonucleoside triphosphate binding	F	2.80E-02	1.61E-04
GO:0006259	DNA metabolic process	P	1.11E-08	2.24E-11
GO:0055114	oxidation-reduction process	P	1.38E-08	3.00E-11
GO:0016491	oxidoreductase activity	F	2.23E-24	1.04E-27
GO:0006310	DNA recombination	P	3.53E-48	9.14E-52
GO:0006518	peptide metabolic process	P	1.36E-02	7.25E-05
GO:0005198	structural molecule activity	F	1.54E-02	8.30E-05
GO:0043604	amide biosynthetic process	P	4.36E-03	2.05E-05
GO:0015074	DNA integration	P	3.34E-77	6.93E-81
GO:0043043	peptide biosynthetic process	P	1.08E-04	3.58E-07
GO:0032196	transposition	P	5.95E-81	6.17E-85
GO:0006412	translation	P	8.85E-05	2.84E-07
GO:0004803	transposase activity	F	2.63E-88	1.36E-92
GO:0006313	transposition, DNA-mediated	P	3.34E-77	6.09E-81
GO:0032259	methylation	P	5.96E-05	1.85E-07
GO:0016741	transferase activity, transferring one-carbon groups	F	2.64E-23	1.51E-26
GO:0008168	methyltransferase activity	F	8.15E-24	4.22E-27
GO:0005840	ribosome	C	2.14E-18	1.66E-21
GO:0016705	oxidoreductase activity, acting on paired donors, with incorporation or reduction of molecular oxygen	F	1.51E-14	2.27E-17
GO:0009123	nucleoside monophosphate metabolic process	P	1.60E-05	4.56E-08
GO:0009161	ribonucleoside monophosphate metabolic process	P	4.69E-06	1.29E-08
GO:1901293	nucleoside phosphate biosynthetic process	P	2.42E-03	1.09E-05
GO:0009167	purine ribonucleoside monophosphate metabolic process	P	2.01E-06	5.10E-09

GO:0009126	purine nucleoside monophosphate metabolic process	P	4.44E-06	1.20E-08
GO:0004386	helicase activity	F	2.48E-04	8.49E-07
GO:0006520	cellular amino acid metabolic process	P	6.84E-03	3.54E-05
GO:0051213	dioxygenase activity	F	4.77E-35	1.98E-38
GO:0003735	structural constituent of ribosome	F	8.37E-15	1.17E-17
GO:0016706	oxidoreductase activity, acting on paired donors, with incorporation or reduction of molecular oxygen, 2-oxoglutarate as one donor, and incorporation of one atom each of oxygen into both donors	F	1.60E-41	4.98E-45
GO:0008135	translation factor activity, RNA binding	F	9.56E-04	3.86E-06
GO:0042254	ribosome biogenesis	P	4.95E-03	2.38E-05
GO:0016853	isomerase activity	F	7.85E-04	3.05E-06
GO:0009124	nucleoside monophosphate biosynthetic process	P	1.56E-14	2.42E-17
GO:0009156	ribonucleoside monophosphate biosynthetic process	P	5.99E-15	8.07E-18
GO:0016779	nucleotidyltransferase activity	F	5.36E-03	2.61E-05
GO:0009168	purine ribonucleoside monophosphate biosynthetic process	P	4.18E-17	3.68E-20
GO:0009127	purine nucleoside monophosphate biosynthetic process	P	6.97E-17	6.50E-20
GO:1905368	peptidase complex	C	3.47E-08	7.90E-11
GO:0006399	tRNA metabolic process	P	9.94E-05	3.25E-07
GO:0016810	hydrolase activity, acting on carbon-nitrogen (but not peptide) bonds	F	2.07E-02	1.12E-04
GO:1905369	endopeptidase complex	C	8.34E-09	1.60E-11
GO:0000502	proteasome complex	C	1.19E-08	2.46E-11
GO:0043044	ATP-dependent chromatin remodeling	P	2.85E-06	7.39E-09
GO:0016651	oxidoreductase activity, acting on NAD(P)H	F	5.63E-04	2.13E-06
GO:1902562	H4 histone acetyltransferase complex	C	2.85E-06	7.52E-09
GO:0090544	BAF-type complex	C	4.08E-09	7.60E-12
GO:0051287	NAD binding	F	2.80E-03	1.28E-05
GO:0019239	deaminase activity	F	3.03E-20	1.88E-23
GO:0043189	H4/H2A histone acetyltransferase complex	C	1.09E-19	7.36E-23
GO:0035267	NuA4 histone acetyltransferase complex	C	1.34E-19	9.70E-23
GO:0016514	SWI/SNF complex	C	7.37E-17	7.25E-20
GO:0016875	ligase activity, forming carbon-oxygen bonds	F	3.17E-04	1.12E-06
GO:0016876	ligase activity, forming aminoacyl-tRNA and related compounds	F	3.17E-04	1.12E-06
GO:0004812	aminoacyl-tRNA ligase activity	F	4.65E-04	1.69E-06
GO:0043039	tRNA aminoacylation	P	1.54E-03	6.72E-06

GO:0043038	amino acid activation	P	1.54E-03	6.72E-06
GO:0043094	cellular metabolic compound salvage	P	1.84E-06	4.59E-09
GO:0006418	tRNA aminoacylation for protein translation	P	8.71E-04	3.48E-06
GO:0015935	small ribosomal subunit	C	2.40E-02	1.36E-04
GO:0016814	hydrolase activity, acting on carbon-nitrogen (but not peptide) bonds, in cyclic amidines	F	2.37E-08	5.27E-11
GO:0038024	cargo receptor activity	F	3.94E-03	1.84E-05
GO:0097525	spliceosomal snRNP complex	C	2.36E-02	1.32E-04
GO:0005044	scavenger receptor activity	F	6.37E-05	2.01E-07
GO:0022624	proteasome accessory complex	C	5.53E-04	2.06E-06
GO:0046040	IMP metabolic process	P	1.25E-08	2.66E-11
GO:0005838	proteasome regulatory particle	C	1.41E-04	4.74E-07
GO:0006188	IMP biosynthetic process	P	1.36E-14	1.97E-17
GO:0043101	purine-containing compound salvage	P	7.37E-08	1.72E-10
GO:0032777	Piccolo NuA4 histone acetyltransferase complex	C	7.20E-18	5.97E-21
GO:0043173	nucleotide salvage	P	1.75E-09	3.08E-12
GO:0005839	proteasome core complex	C	2.02E-03	8.87E-06
GO:0003950	NAD+ ADP-ribosyltransferase activity	F	4.74E-03	2.26E-05
GO:0016917	GABA receptor activity	F	2.01E-09	3.64E-12
GO:0008540	proteasome regulatory particle, base subcomplex	C	2.13E-07	5.19E-10
GO:0004298	threonine-type endopeptidase activity	F	6.02E-03	2.99E-05
GO:0070003	threonine-type peptidase activity	F	6.02E-03	2.99E-05
GO:0032264	IMP salvage	P	2.02E-13	3.24E-16
GO:0032261	purine nucleotide salvage	P	1.65E-12	2.74E-15
GO:0004890	GABA-A receptor activity	F	2.22E-11	3.79E-14
GO:0047623	adenosine-phosphate deaminase activity	F	2.89E-15	3.14E-18
GO:0003876	AMP deaminase activity	F	2.89E-15	3.14E-18
GO:0003964	RNA-directed DNA polymerase activity	F	5.24E-04	1.93E-06
GO:0080025	phosphatidylinositol-3,5-bisphosphate binding	F	1.05E-03	4.37E-06
GO:0048038	quinone binding	F	1.70E-07	4.06E-10
GO:0045259	proton-transporting ATP synthase complex	C	1.52E-03	6.47E-06
GO:0016854	racemase and epimerase activity	F	4.57E-05	1.40E-07
GO:0034030	ribonucleoside bisphosphate biosynthetic process	P	6.11E-03	3.14E-05
GO:0034033	purine nucleoside bisphosphate biosynthetic process	P	6.11E-03	3.14E-05
GO:0033866	nucleoside bisphosphate biosynthetic process	P	6.11E-03	3.14E-05
GO:0018112	proline racemase activity	F	3.95E-15	5.11E-18

GO:0016855	racemase and epimerase activity, acting on amino acids and derivatives	F	3.95E-15	5.11E-18
GO:0047661	amino-acid racemase activity	F	3.95E-15	5.11E-18
GO:0036361	racemase activity, acting on amino acids and derivatives	F	3.95E-15	5.11E-18
GO:0015937	coenzyme A biosynthetic process	P	3.01E-03	1.39E-05
GO:0045263	proton-transporting ATP synthase complex, coupling factor F(o)	C	8.17E-03	4.27E-05
GO:0022833	mechanically gated channel activity	F	3.02E-02	1.77E-04
GO:0008381	mechanically-gated ion channel activity	F	3.02E-02	1.77E-04
GO:0004594	pantothenate kinase activity	F	1.26E-03	5.27E-06
GO:0005753	mitochondrial proton-transporting ATP synthase complex	C	3.53E-02	0.000211
GO:0015016	[heparan sulfate]-glucosamine N-sulfotransferase activity	F	2.34E-02	1.30E-04
GO:0008418	protein-N-terminal asparagine amidohydrolase activity	F	6.76E-04	2.59E-06
GO:0004035	alkaline phosphatase activity	F	8.35E-04	3.29E-06
GO:0004649	poly(ADP-ribose) glycohydrolase activity	F	9.63E-04	3.94E-06
GO:0004936	alpha-adrenergic receptor activity	F	2.34E-02	1.30E-04
GO:0004937	alpha1-adrenergic receptor activity	F	1.18E-02	6.25E-05
