# Supplementary material for: De novo assembly of the sea trout (Salmo trutta m. trutta) skin transcriptome to identify putative genes involved in the immune response and epidermal mucus secretion
Source: PLoS One. 2017 Feb 17;12(2):e0172282. doi: 10.1371/journal.pone.0172282 (PMC5315281; doi:10.1371/journal.pone.0172282)
Supplement: S4 Table — (PDF) [file pone.0172282.s007.pdf]

S4 Table. Classification of the mucins identified in multi-tissue transcriptome, according to the BLASTX and BLASTN searches.

**Contig**	**Size (nt)**	**Annotation (NR)**	**Accession number (NR)**	**E-value**	***Salmo salar* genome (ID)**
ST_91149	419	*S.salar* , MUC2	XP_014040158	1.00E-84	NW_012349946.1
ST_88998	787	*S.salar* , MUC2	XP_014025861	7.00E-164	NC_027322.1
ST_4188	1900	*S.salar* , MUC2	XP_013980031	0	NC_027309.1
ST_6301	3702	*S.salar* , MUC2	XP_014025397	0	NC_027322.1
ST_42208	796	*S.salar* , MUC2	XP_014061905	2.00E-32	NC_027306.1
ST_5641	1452	*S.salar* , MUC5AC	XP_014036802	0	NC_027310.1
ST_6179	1314	*S.salar* , MUC5B	XP_014031349	5.00E-116	NC_027325.1
ST_76849	478	*S.salar* , MUC5AC	XP_013996218	2.00E-83	NC_027313.1
ST_9981	2060	*S.salar* , MUC5B	XP_014031349	0	NC_027325.1
ST_10480	2868	*S.salar* , I-MUC	XP_014041914	0	NC_027309.1
ST_23799	4525	*S.salar* , I-MUC	XP_014031401	0	NW_012360345.1
ST_27584	2729	*S.salar* , I-MUC	XP_014038548	0	NW_012355657.1
ST_27547	734	*S.salar* , MUC13	XP_014014132	1.00E-92	NC_027318.1
ST_28361	2762	*S.salar* , MUC17	XP_014011797	0	NC_027317.1
