# Supplementary material for: De novo assembly of the sea trout (Salmo trutta m. trutta) skin transcriptome to identify putative genes involved in the immune response and epidermal mucus secretion
Source: PLoS One. 2017 Feb 17;12(2):e0172282. doi: 10.1371/journal.pone.0172282 (PMC5315281; doi:10.1371/journal.pone.0172282)
Supplement: S5 Table — (PDF) [file pone.0172282.s008.pdf]

S5 Table. Genes involved in mucin biosynthesis and mucus production in sea trout skin transcriptome.

Functional group	Gene Symbol
**Mucins**	*I-Muc, Muc2, Muc5B, Muc5AC, Muc12, Muc13, Muc15, Muc17*
**PDI**	*PDIA1, PDIA3, PDIA4, PDIA6, AGR2*
**Glycosyltransferases**	*GALNT2, GALNT3, GALNT4, GALNT5, GALNT6, GALNT7, GALNT8, GALNT12, GALNT14, GALNT15, GALNT18, C1GALT1, GCNT3, GCNT4, C1GALT2, C1GALT1C1, B3GNT1, B3GNT2, B3GNT3, B3GNT4, B3GALT1, B3GALT2, B4GALT1, B4GALT3, B4GALT7, ST6GALNAC4, ST6GALNAC5A, ST6GALNAC1, ST3GAL3, ST3GAL1, ST3GAL8, ST8SIA5, ST8SIA6, ST8SIA7, CHST2, CHST3, CHST7, CHST10, CHST12, CHST14, CHST15, FUT1, FUT9, FUT10, FUT11*
**Transcription factors**	*SPDEF*
**Receptors**	*EGFR*
**RAB**	*RAB1A, RAB1B, RAB2A, RAB2B, RAB2C, RAB3D, RAB4B, RAB5A, RAB5C, RAB6A, RAB6B, RAB7A, RAB8A, RAB9A, RAB10, RAB11A, RAB11B, RAB12, RAB13, RAB14, RAB18, RAB19, RAB20, RAB21, RAB22A, RAB24, RAB25, RAB27A, RAB27B, RAB31, RAB32, RAB35, RAB37, RAB39B, RAB40B, RAB41*
**SNARE**	*STX1B, STX3, STX4, STX5, STX6, STX7, STX8, STX10, STX11, STX12, STX16, STX17, STX18, VAMP2, VAMP3, VAMP5, VAMP7, VAMP8*
**Galectins**	*LGALS1, LGALS3*
**Aquaporins**	*AQP1, AQP3, AQP11, AQP12*
**Ion channels, ion pumps and transporters**	*ATP1A1, ATP1A3, ATP1B3, SLC12A2, ATP2A1. SCNN1A, ATP1B1, ATP2A2, ATP2A3, ATP1A2, ATP1B1, ATP2B3, CLCA1*
**Regulation factors**	*MARCKS, PKCβ, PKCι, PKCθ, PKCδ, PKCη, PKCε*

Genes are abbreviated as Muc: mucin; PDIA: protein disulfide isomerase family A; GALNT: Polypeptide N-acetylgalactosaminyltransferase; C1GALT: Glycoprotein-N-acetylgalactosamine 3-beta-galactosyltransferase; GCNT: Beta-1,3-galactosyl-O-glycosyl-glycoprotein beta-1,6-N-acetylglucosaminyltransferase; B3GNT: UDP-GlcNAc:betaGal beta-1,3-N-acetylglucosaminyltransferase; B3GALT: beta-1,3-galactosyltransferase; ST6GALNAC: N-acetylgalactosaminide alpha-2,6-sialyltransferase; ST3GAL: Beta-Galactoside Alpha-2,3-Sialyltransferase; CHST: Carbohydrate Sulfotransferase; FUT-1: GDP-fucose transporter 1; SPDEF: SAM Pointed Domain Containing ETS Transcription Factor; EGFR: Epidermal growth factor receptor; RAB: Ras-related protein; STX: Syntaxin; VAMP: Vesicle-associated membrane protein; LGASL: Lectin, Galactoside-Binding, Soluble; AQP: Aquaporin; ATP1A: Sodium/potassium-transporting ATPase subunit alpha; ATP1B: Sodium/potassium-transporting ATPase subunit beta; SCNN: Sodium Channel, Non Voltage Gated; CLCA: Chloride Channel Accessory;SLC: Solute carrier family; ATP2A: Sarcoplasmic/endoplasmic reticulum calcium ATPase 1. MARCKS: Myristoylated alanine-rich C-kinase substrate; PKC: Protein kinase C.

PDI: folding and oligomerization of mucins [1]. Glycotransferases: glycosylations by adding the initial N-acetyl galactosamine (GalNAc) to a serine/threonine residue on the apomucin, forming the core structure of an O-glycan, forming the glycan's lactosamine or polylactosamine backbone and adding the glycan's peripheral structure [2]. RAB and SNARE: vesicle trafficking and mediate to fusion of carrier vesicle with the target membrane [3]. Galectins: associated with mucin binding [4]. Aquaporins: regulation of transcellular and paracellular water movement [5]. Ion channels, ion pumps and transporters: hydration, concentration of calcium [6, 7].

Supporting References

1. Park SW, Zhen G, Verhaeghe C, Nakagami Y, Nguyenvu LT, Barczak AJ, et al. The protein disulfide isomerase AGR2 is essential for production of intestinal mucus. Proc Natl Acad Sci. 2009; 106(17): 6950-6955.

2. Fahy JV, Dickey BF. Airway mucus function and dysfunction. N Engl J Med. 2010; 363(23): 2233-2247.

3. Kim M, Kim S, Kim H, Joo HG, Shin T. Immunohistochemical localization of galectin-3 in the reproductive organs of the cow. Acta Histochem. 2008; 110(6): 473-480.

4. Kwong RWM, Kumai Y, Perry SF. The role of aquaporin and tight junction proteins in the regulation of water movement in larval zebrafish (*Danio rerio*). PLoS ONE. 2013; 8(8): e70764.

5. Muchekehu RW, Quinton PM. A new role for bicarbonate secretion in cervico uterine mucus release. J Physiol. 2010; 588: 2329-2342.

6. Patsos G, Corfield A. O-Glycosylation: structural diversity and functions. In: Gabius HJ, editor. The Sugar Code: Fundamentals of Glycosciences. Weinheim: Wiley-VCH; 2009. pp. 111-137.

7. Pluta K, McGettigan PA, Reid CJ, Browne JA, Irwin JA, Tharmalingam T, et al. Molecular aspects of mucin biosynthesis and mucus formation in the bovine cervix during the periestrous period. Physiol Genomics. 2012; 44(24): 1165-78.
