# Supplementary material for: De novo assembly of the sea trout (Salmo trutta m. trutta) skin transcriptome to identify putative genes involved in the immune response and epidermal mucus secretion
Source: PLoS One. 2017 Feb 17;12(2):e0172282. doi: 10.1371/journal.pone.0172282 (PMC5315281; doi:10.1371/journal.pone.0172282)
Supplement: S6 Table — (PDF) [file pone.0172282.s009.pdf]

S6 Table. List of immunity-related genes found in sea trout skin transcriptome.

Gene symbol	Description
**A1AT**	serpin peptidase inhibitor, clade A (alpha-1 antiproteinase, antitrypsin), member 1
**A2M**	alpha-2-macroglobulin
**ABL2**	ABL proto-oncogene 2, non-receptor tyrosine kinase
**ACTG1**	actin gamma 1
**ACTN1**	actinin alpha 1
**ACTN2**	actinin alpha 2
**ACTR1B**	ARP1 actin-related protein 1 homolog B, centractin beta (yeast)
**ACTR2**	ARP2 actin-related protein 2 homolog (yeast)
**ACTR3**	ARP3 actin-related protein 3 homolog (yeast)
**ADA**	adenosine deaminase
**ADAM10**	ADAM metallopeptidase domain 10
**ADAR**	adenosine deaminase, RNA-specific
**ADCY2**	adenylate cyclase 2 (brain)
**ADCY4**	adenylate cyclase 4
**ADCY6**	adenylate cyclase 6
**ADCY8**	adenylate cyclase 8 (brain)
**ADIPOQ**	adiponectin, C1Q and collagen domain containing
**ADNP**	activity-dependent neuroprotector homeobox
**ADNP2**	ADNP homeobox 2
**ADRM1**	adhesion regulating molecule 1
**AGO4**	argonaute 4, RISC catalytic component
**AGPAT5**	1-acylglycerol-3-phosphate O-acyltransferase 5
**AIFM2**	apoptosis inducing factor, mitochondria associated 2
**AIFM3**	apoptosis inducing factor, mitochondria associated 3
**AK2**	adenylate kinase 2
**AKT1**	v-akt murine thymoma viral oncogene homolog 1
**ALAS2**	5'-aminolevulinate synthase 2
**ALP**	atherosclerosis susceptibility (lipoprotein associated)
**AMPD3**	adenosine monophosphate deaminase 3
**AMPH**	amphiphysin
**ANGPT1**	angiopoietin 1
**ANO6**	anoctamin 6
**ANPEP**	alanyl aminopeptidase, membrane
**ANXA11**	annexin A11
**AP1B1**	adaptor related protein complex 1 beta 1 subunit
**AP1M1**	adaptor related protein complex 1 mu 1 subunit
**AP1S1**	adaptor related protein complex 1 sigma 1 subunit
**AP2A2**	Adaptor-Related Protein Complex 2, Alpha 2 Subunit
**AP3D1**	Adaptor-Related Protein Complex 3, Delta 1 Subunit
**APBB1**	amyloid beta precursor protein binding family B member 1
**APC**	adenomatous polyposis coli
**ARAF**	A-Raf proto-oncogene, serine/threonine kinase
**ARF1**	ADP ribosylation factor 1
**ARF6**	ADP ribosylation factor 6
**ARHGAP5**	Rho GTPase activating protein 5
**ARHGEF1**	Rho guanine nucleotide exchange factor 1
**ARHGEF12**	Rho guanine nucleotide exchange factor 12

**ARID4A**	AT-rich interaction domain 4A
**ARIH2**	ariadne RBR E3 ubiquitin protein ligase 2
**ARPC1A_B**	actin related protein 2/3 complex, subunit 1A/1B
**ARPC2**	actin related protein 2/3 complex subunit 2
**ARPC3**	actin related protein 2/3 complex subunit 3
**ARPC4**	actin related protein 2/3 complex subunit 4
**ARPC5**	actin related protein 2/3 complex subunit 5
**ARRB1**	arrestin, beta 1
**ASAP1**	ArfGAP with SH3 domain, ankyrin repeat and PH domain 1
**ATG101**	autophagy related 101
**ATG12**	autophagy related 12
**ATG13**	autophagy related 13
**ATG16L1**	autophagy related 16 like 1
**ATG2A**	autophagy related 2A
**ATG2B**	autophagy related 2B
**ATG3**	autophagy related 3
**ATG5**	autophagy related 5
**ATG9A**	autophagy related 9A
**ATIII**	serpin peptidase inhibitor, clade C (antithrombin), member 1
**B4GALT1**	beta-1,4-galactosyltransferase 1
**BATF**	basic leucine zipper ATF-like transcription factor
**BCL10**	B-cell CLL/lymphoma 10
**BCL11B**	B-cell CLL/lymphoma 11B
**BCL6B**	B-cell CLL/lymphoma 6B
**BCL7A**	B-cell CLL/lymphoma 7A
**BCL7B**	B-cell CLL/lymphoma 7B
**BCL9**	B-cell CLL/lymphoma 9
**BIRC3**	baculoviral IAP repeat containing 3
**BLNK**	B-cell linker
**BMI1**	BMI1 proto-oncogene, polycomb ring finger
**BNIP3**	BCL2/adenovirus E1B 19kDa interacting protein 3
**BPI**	bactericidal/permeability-increasing protein
**BRD2**	bromodomain containing 2
**BRD3**	bromodomain containing 3
**BRD4**	bromodomain containing 4
**BRK1**	BRICK1, SCAR/WAVE actin-nucleating complex subunit
**BTK**	Bruton tyrosine kinase
**BTL**	cysteine rich hydrophobic domain 2
**BTN1A1**	butyrophilin subfamily 1 member A1
**C1QA**	complement component 1, q subcomponent, A chain
**C1QB**	complement component 1, q subcomponent, B chain
**C1QBP**	complement component 1, q subcomponent binding protein
**C1QC**	complement component 1, q subcomponent, C chain
**C1RL**	complement C1r subcomponent like
**C1S**	complement component 1, s subcomponent
**C3**	complement component 3
**C3AR1**	complement component 3a receptor 1
**C4**	complement component 4A (Rodgers blood group)
**C5**	complement component 5
**C6**	complement component 6

**C7**	complement component 7
**C8B**	Complement Component 8, Beta Polypeptide
**CALM1**	calmodulin 1 (phosphorylase kinase, delta)
**CALR**	calreticulin
**CANX**	calnexin
**CAPZA1**	capping actin protein of muscle Z-line alpha subunit 1
**CARD11**	caspase recruitment domain family member 11
**CARD9**	caspase recruitment domain family member 9
**CASP1**	caspase 1
**CASP3**	caspase 3
**CASP8**	caspase 8
**CATH-1A**	cathelicidin-derived antimicrobial peptide 1 isoform a
**CATH2**	cathelicidin 2
**CAV1**	caveolin 1
**CBFB**	core-binding factor, beta subunit
**CBL**	Cbl proto-oncogene, E3 ubiquitin protein ligase
**CCL13**	C-C motif chemokine ligand 13
**CCL19**	C-C motif chemokine ligand 19
**CCL2**	C-C motif chemokine ligand 2
**CCL20**	C-C motif chemokine ligand 20
**CCL21**	C-C motif chemokine ligand 21
**CCL25**	C-C motif chemokine ligand 25
**CCL28**	C-C motif chemokine ligand 28
**CCL3**	C-C motif chemokine ligand 3
**CCL34B.4**	Chemokine (C-C motif) ligand 34b, duplicate 4
**CCL4**	C-C motif chemokine ligand 4
**CCL8**	C-C motif chemokine ligand 8
**CCR1**	C-C motif chemokine receptor 1
**CCR3**	C-C motif chemokine receptor 3
**CCR4**	C-C motif chemokine receptor 4
**CCR6**	C-C motif chemokine receptor 6
**CCR7**	C-C motif chemokine receptor 7
**CCR9**	C-C motif chemokine receptor 9
**CD029**	Chromosome 4 Open Reading Frame 29
**CD032**	Chromosome 4 Open Reading Frame 32
**CD151**	CD151 molecule (Raph blood group)
**CD160**	CD160 molecule
**CD163**	CD163 molecule
**CD166**	activated leukocyte cell adhesion molecule
**CD2**	CD2 molecule
**CD209A**	cd209 antigen-like protein a
**CD209B**	cd209 antigen-like protein b
**CD209C**	cd209 antigen-like protein c
**CD209D**	cd209 antigen-like protein d
**CD209E**	cd209 antigen-like protein e
**CD22**	CD22 molecule
**CD276**	CD276 molecule
**CD2BP2**	CD2 (cytoplasmic tail) binding protein 2
**CD302**	CD302 molecule
**CD34A**	cd34a molecule

**CD4-2B**	cd4-2b-like protein
**CD44**	CD44 molecule
**CD46**	CD46 molecule
**CD48**	CD48 molecule
**CD55**	CD55 molecule
**CD59**	CD59 molecule
**CD63**	CD63 molecule
**CD74**	CD74 molecule
**CD80**	CD80 molecule
**CD81**	CD81 molecule
**CD82**	CD82 molecule
**CD83**	CD83 molecule
**CD8A**	CD8a molecule
**CD8B**	CD8b molecule
**CD9**	CD9 molecule
**CD97**	adhesion G protein-coupled receptor E5
**CD99**	CD99 molecule
**CDC37**	cell division cycle 37
**CDC42**	cell division cycle 42
**CDC73**	cell division cycle 73
**CDH5**	cadherin 5
**CEBPA**	CCAAT/enhancer binding protein alpha
**CEBPG**	CCAAT/enhancer binding protein gamma
**CFB**	complement factor B
**CFD**	complement factor D (adipsin)
**CFH**	complement factor H
**CFL1**	cofilin 1
**Cgas**	Mab-21 domain containing 1
**CHUK**	conserved helix-loop-helix ubiquitous kinase
**CIAPIN1**	cytokine induced apoptosis inhibitor 1
**CIQL2**	complement c1q-like protein 2
**CIQL4**	complement c1q-like protein 4
**CITED2**	Cbp/p300 interacting transactivator with Glu/Asp rich carboxy-terminal domain 2
**CK-1**	chemokine ck-1
**CLDN1**	claudin 1
**CLEC10A**	C-type lectin domain family 10 member A
**CLEC11A**	C-type lectin domain family 11 member A
**CLEC14A**	C-type lectin domain family 14 member A
**CLEC18A**	C-type lectin domain family 18 member A
**CLEC4E**	C-type lectin domain family 4 member E
**CLEC4F**	C-type lectin domain family 4 member F
**CLEC4M**	C-type lectin domain family 4 member M
**CLPX**	caseinolytic mitochondrial matrix peptidase chaperone subunit
**CLTC**	clathrin heavy chain
**CNIH1**	cornichon family AMPA receptor auxiliary protein 1
**CNPY3**	canopy FGF signaling regulator 3
**COLEC12**	collectin subfamily member 12
**CORO1A**	coronin 1A
**CPSF1**	cleavage and polyadenylation specific factor 1
**CR1**	complement component 3b/4b receptor 1 (Knops blood group)

**CR2L**	complement receptor type 2
**CREB**	creb-binding
**CRIP2**	cysteine rich protein 2
**CRK**	v-crk avian sarcoma virus CT10 oncogene homolog
**CSF1R**	colony stimulating factor 1 receptor
**CSF3**	colony stimulating factor 3
**CSK**	c-src tyrosine kinase
**CSL2**	l-rhamnose-binding lectin csl2-like
**CTLA-4**	cytotoxic T-lymphocyte-associated protein 4
**CTNNA1**	catenin alpha 1
**CTNNB1**	catenin beta 1
**CTNND1**	catenin delta 1
**CTSB**	cathepsin B
**CTSC**	cathepsin C
**CTSD**	cathepsin D
**CTSF**	cathepsin F
**CTSK**	cathepsin K
**CTSL**	cathepsin L
**CTSO**	cathepsin O
**CTSS**	cathepsin S
**CTSZ**	cathepsin Z
**CUL1**	cullin 1
**CUL3**	cullin 3
**CUL4A**	cullin 4A
**CXCD1**	C-X-C chemokine d1
**CXCL12**	C-X-C motif chemokine ligand 12
**CXCL14**	C-X-C motif chemokine ligand 14
**CXCL6**	C-X-C motif chemokine ligand 6
**CXCR3**	C-X-C motif chemokine receptor 3
**CXCR4**	C-X-C motif chemokine receptor 4
**CXXC1**	CXXC finger protein 1
**CYBA**	cytochrome b-245 alpha chain
**CYFIP1**	cytoplasmic FMR1 interacting protein 1
**CYLD**	CYLD lysine 63 deubiquitinase
**DAN4**	cell wall protein dan4
**DCTN5**	dynactin subunit 5
**DDOST**	dolichyl-diphosphooligosaccharide--protein glycosyltransferase non-catalytic subunit
**DDX1**	DEAD/H-box helicase 1
**DDX18**	DEAD-box helicase 18
**DDX23**	DEAD-box helicase 23
**DDX3X**	DEAD-box helicase 3, X-linked
**DDX41**	DEAD-box helicase 41
**DDX58**	DEXD/H-box helicase 58
**DEFB1**	defensin beta 1
**DHX58**	DEXH-box helicase 58
**DKC1**	dyskerin pseudouridine synthase 1
**DLG1**	discs large homolog 1, scribble cell polarity complex component
**DNAJA3**	DnaJ heat shock protein family (Hsp40) member A3
**DNM2**	dynamin 2
**DNTT**	DNA nucleotidylexotransferase

**DOCK1**	dedicator of cytokinesis 1
**DOCK2**	dedicator of cytokinesis 2
**DOCK7**	dedicator of cytokinesis 7
**DYNC1I2**	dynein cytoplasmic 1 intermediate chain 2
**DYNLL1**	dynein light chain LC8-type 1
**ECSIT**	ECSIT signalling integrator
**EDAR**	ectodysplasin A receptor
**EDNRB**	endothelin receptor type B
**EEF2**	eukaryotic translation elongation factor 2
**EFNA2**	ephrin-A2
**EFNA4**	ephrin-A4
**EIF2AK2**	eukaryotic translation initiation factor 2 alpha kinase 2
**ELMO1**	engulfment and cell motility 1
**EMILIN1**	elastin microfibril interfacer 1
**ENDOU**	endonuclease, poly(U) specific
**ENPP1**	ectonucleotide pyrophosphatase/phosphodiesterase 1
**ENPP2**	ectonucleotide pyrophosphatase/phosphodiesterase 2
**EOMES**	eomesodermin
**EPHX2**	epoxide hydrolase 2
**EPPK1**	epiplakin 1
**ERAP1**	endoplasmic reticulum aminopeptidase 1
**ERAP2**	endoplasmic reticulum aminopeptidase 2
**ERBB2IP**	erbb2 interacting protein
**ERCC2**	excision repair cross-complementation group 2
**ETNPPL**	ethanolamine-phosphate phospho-lyase
**ETS1**	ETS proto-oncogene 1, transcription factor
**ETV5**	ETS variant 5
**ETV7**	ETS variant 7
**EXOC6**	exocyst complex component 6
**EZR**	ezrin
**F13A1**	coagulation factor XIII A chain
**F3**	coagulation factor III, tissue factor
**F5**	coagulation factor V
**F8**	coagulation factor VIII
**FADD**	Fas associated via death domain
**FAM20C**	family with sequence similarity 20 member C
**FBXO5**	F-box protein 5
**FCER1G**	Fc fragment of IgE receptor Ig
**FER**	FER tyrosine kinase
**FERMT3**	fermitin family member 3
**FEV**	FEV, ETS transcription factor
**FGFR1**	fibroblast growth factor receptor 1
**FGFR2**	fibroblast growth factor receptor 2
**FGR**	FGR proto-oncogene, Src family tyrosine kinase
**FKBP1B**	FK506 binding protein 1B
**FOS**	FBJ murine osteosarcoma viral oncogene homolog
**FOXO3**	forkhead box O3
**FRK**	fyn related Src family tyrosine kinase
**FTL**	ferritin, light polypeptide
**FYB**	FYN binding protein

**FYN**	FYN proto-oncogene, Src family tyrosine kinase
**G6PD**	glucose-6-phosphate dehydrogenase
**GAR1**	GAR1 ribonucleoprotein
**GATA2**	GATA binding protein 2
**GBF1**	golgi brefeldin A resistant guanine nucleotide exchange factor 1
**GBP1**	guanylate binding protein 1
**GFI1**	growth factor independent 1 transcription repressor
**GFI1B**	growth factor independent 1B transcription repressor
**GIG1**	natural killer cell granule protein 7
**GIG2**	tribbles pseudokinase 1
**GIMAP7**	GTPase, IMAP family member 7
**GLG1**	golgi glycoprotein 1
**GLRX5**	glutaredoxin 5
**GNAI1**	G protein subunit alpha i1
**GNAQ**	G protein subunit alpha q
**GNAS**	GNAS complex locus
**GNB1**	G protein subunit beta 1
**GNG10**	G protein subunit gamma 10
**GNG12**	G protein subunit gamma 12
**GNG13**	G protein subunit gamma 13
**GNG2**	G protein subunit gamma 2
**GNG5**	G protein subunit gamma 5
**GNG7**	G protein subunit gamma 7
**GOLPH3**	golgi phosphoprotein 3
**GPR183**	G protein-coupled receptor 183
**GRAP2**	GRB2-related adaptor protein 2
**GRK**	G protein-coupled receptor kinase
**GRLF1**	Rho GTPase activating protein 35
**GSK3B**	glycogen synthase kinase 3 beta
**GSN**	gelsolin
**GVIN1**	GTPase, very large interferon inducible pseudogene 1
**GZMA**	granzyme A
**GZMB**	granzyme B
**GZMG**	granzyme G
**GZMK**	granzyme K
**H2A**	histone cluster 2, H2ac
**H2-M2**	Murine MHC class Ib
**H2-Q10**	H-2 class I histocompatibility antigen, Q10 alpha chain
**HCK**	HCK proto-oncogene, Src family tyrosine kinase
**HECTD1**	HECT domain E3 ubiquitin protein ligase 1
**HES1**	hes family bHLH transcription factor 1
**HGPRT**	hypoxanthine phosphoribosyltransferase 1
**HHEX**	hematopoietically expressed homeobox
**HLA-A**	major histocompatibility complex, class I, A
**HLA-F**	major histocompatibility complex, class I, F
**HRAS**	Harvey rat sarcoma viral oncogene homolog
**HRG1**	solute carrier family 48 member 1
**HSBP1**	heat shock factor binding protein 1
**Hsc70-5**	heat shock 70 kda protein cognate 5
**HSF1**	heat shock transcription factor 1

**HSF2**	heat shock transcription factor 2
**HSP30**	heat shock protein 30
**HSP70**	heat shock protein 70
**HSP75**	TNF receptor-associated protein 1
**HSP90**	heat shock protein 90kDa alpha family class A member 1
**HSP90AA1**	Heat Shock Protein 90kDa Alpha (Cytosolic), Class A Member 1
**HSP90AB1**	Heat Shock Protein 90kDa Alpha (Cytosolic), Class B Member 1
**HSPA1**	heat shock protein family A (Hsp70) member 1A
**HSPA13**	heat shock protein family A (Hsp70) member 13
**HSPA14**	heat shock protein family A (Hsp70) member 14
**HSPA4**	heat shock protein family A (Hsp70) member 4
**HSPA4L**	heat shock protein family A (Hsp70) member 4 like
**HSPA8**	heat shock protein family A (Hsp70) member 8
**HSPA9**	heat shock protein family A (Hsp70) member 9
**HSPB1**	heat shock protein family B (small) member 1
**HSPB2**	heat shock protein family B (small) member 2
**HSPB6**	heat shock protein family B (small) member 6
**HSPB7**	heat shock protein family B (small) member 7
**HSPB8**	heat shock protein family B (small) member 8
**HSPD1**	heat shock protein family D (Hsp60) member 1
**HTPG**	Chaperone protein HtpG
**IFI30**	interferon gamma inducible protein 30
**IFI35**	interferon induced protein 35
**IFI44**	interferon induced protein 44
**IFIH1**	interferon induced with helicase C domain 1
**IFIT1B**	interferon induced protein with tetratricopeptide repeats 1B
**IFIT2**	interferon induced protein with tetratricopeptide repeats 2
**IFIT5**	interferon induced protein with tetratricopeptide repeats 5
**IFNA2**	interferon, alpha 2
**IFNAR**	interferon alpha and beta receptor subunit 1
**IFNAR2**	interferon alpha and beta receptor subunit 2
**IFNGR1**	interferon gamma receptor 1
**IFNGR2**	interferon gamma receptor 2 (interferon gamma transducer 1)
**IFRD1**	interferon related developmental regulator 1
**IFRD2**	interferon-related developmental regulator 2
**IGBP1**	immunoglobulin (CD79A) binding protein 1
**IGFN1**	immunoglobulin-like and fibronectin type III domain containing 1
**IGHD**	immunoglobulin heavy constant delta
**IGHM**	immunoglobulin heavy constant mu
**IGSF10**	immunoglobulin superfamily member 10
**IGSF6**	immunoglobulin superfamily member 6
**IKBKB**	inhibitor of kappa light polypeptide gene enhancer in B-cells, kinase beta
**IKBKE**	inhibitor of kappa light polypeptide gene enhancer in B-cells, kinase epsilon
**IKBKG**	inhibitor of kappa light polypeptide gene enhancer in B-cells, kinase gamma
**IL10RB**	interleukin 10 receptor subunit beta
**IL13RA2**	interleukin 13 receptor subunit alpha 2
**IL15**	interleukin 15
**IL17F**	interleukin 17F
**IL17RA**	interleukin 17 receptor A
**IL17RB**	interleukin 17 receptor B

**IL17RD**	interleukin 17 receptor D
**IL18**	interleukin 18
**IL1B**	interleukin 1 beta
**IL1R2**	Interleukin 1 Receptor, Type II
**IL1R1**	Interleukin 1 Receptor, Type I
**IL1RAP**	interleukin 1 receptor accessory protein
**IL1RN**	interleukin 1 receptor antagonist
**IL2RG**	Interleukin 2 Receptor, Gamma
**IL2RB**	interleukin 2 receptor subunit beta
**IL34**	interleukin 34
**IL4R**	interleukin 4 receptor
**IL5RA**	interleukin 5 receptor subunit alpha
**IL6R**	interleukin 6 receptor
**IL6RA**	interleukin 6 receptor subunit alpha
**IL6RB**	interleukin 6 receptor subunit beta
**IL7RA**	interleukin 7 receptor subunit alpha
**IL8RA**	C-X-C motif chemokine receptor 1
**ILDR1**	immunoglobulin like domain containing receptor 1
**ILF2**	interleukin enhancer binding factor 2
**ILF3**	interleukin enhancer binding factor 3
**IMPDH1a**	IMP (inosine 5'-monophosphate) dehydrogenase 1
**IMPDH2**	IMP (inosine 5'-monophosphate) dehydrogenase 2
**INPP5D**	inositol polyphosphate-5-phosphatase D
**INPPL1**	inositol polyphosphate phosphatase like 1
**IRAK1**	interleukin 1 receptor associated kinase 1
**IRAK3**	interleukin 1 receptor associated kinase 3
**IRAK4**	interleukin 1 receptor associated kinase 4
**IRF1**	interferon regulatory factor 1
**IRF2**	interferon regulatory factor 2
**IRF2BP1**	interferon regulatory factor 2 binding protein 1
**IRF2BP2**	interferon regulatory factor 2 binding protein 2
**IRF2BP2A**	Interferon Regulatory Factor 2 Binding Protein 2A
**IRF2BP2B**	Interferon Regulatory Factor 2 Binding Protein 2B
**IRF3**	interferon regulatory factor 3
**IRF4**	interferon regulatory factor 4
**IRF5**	interferon regulatory factor 5
**IRF6**	interferon regulatory factor 6
**IRF7**	interferon regulatory factor 7
**IRF8**	interferon regulatory factor 8
**IRF9**	interferon regulatory factor 9
**IRGC1**	immunity-related GTPase family, cinema
**ISG20L2**	interferon stimulated exonuclease gene 20kDa like 2
**ITCH**	itchy E3 ubiquitin protein ligase
**ITGA2**	integrin subunit alpha 2
**ITGA3**	integrin subunit alpha 3
**ITGA4**	integrin subunit alpha 4
**ITGA5**	integrin subunit alpha 5
**ITGA6**	integrin subunit alpha 6
**ITGB1**	integrin subunit beta 1
**ITGB2**	integrin subunit beta 2

**ITGB3**	integrin subunit beta 3
**ITGB7**	integrin subunit beta 7
**ITPR1**	inositol 1,4,5-trisphosphate receptor type 1
**ITPR2**	inositol 1,4,5-trisphosphate receptor type 2
**ITPR3**	inositol 1,4,5-trisphosphate receptor type 3
**JAG1**	jagged 1
**JAGN1B**	Jagunal Homolog 1B
**JAK1**	Janus kinase 1
**JAK2**	Janus kinase 2
**JAM1**	F11 receptor
**JAM2**	junctional adhesion molecule 2
**JAM3**	junctional adhesion molecule 3
**JARID2**	jumonji and AT-rich interaction domain containing 2
**JNK**	mitogen-activated protein kinase 8
**JUN**	jun proto-oncogene
**KALRN**	kalirin, RhoGEF kinase
**KDM1A**	lysine demethylase 1A
**KLC1**	kinesin light chain 1
**KLF1**	Kruppel-like factor 1 (erythroid)
**KLKB1**	kallikrein B1
**KMT2A**	lysine methyltransferase 2A
**KRAS**	Kirsten rat sarcoma viral oncogene homolog
**LAMTOR2**	late endosomal/lysosomal adaptor, MAPK and MTOR activator 2
**LAMTOR3**	late endosomal/lysosomal adaptor, MAPK and MTOR activator 3
**LBP**	lipopolysaccharide binding protein
**LCLAT1**	lysocardiolipin acyltransferase 1
**LCP1**	lymphocyte cytosolic protein 1 (L-plastin)
**LCP2**	lymphocyte cytosolic protein 2
**LDB1**	LIM domain binding 1
**LEF1**	lymphoid enhancer binding factor 1
**LGALS1**	lectin, galactoside-binding, soluble, 1
**LGALS3**	lectin, galactoside-binding, soluble, 3
**LGALS4**	lectin, galactoside-binding, soluble, 4
**LGALS8**	lectin, galactoside-binding, soluble, 8
**LGALS9**	lectin, galactoside-binding, soluble, 9
**LGMN**	legumain
**LIG4**	ligase IV, DNA, ATP-dependent
**LIMK1**	LIM domain kinase 1
**LIMK2**	LIM domain kinase 2
**PAFAH1B1**	Platelet-Activating Factor Acetylhydrolase 1b, Regulatory Subunit 1 (45kDa)
**LMO4**	LIM domain only 4
**LNPEP**	leucyl/cystinyl aminopeptidase
**LPIN1**	lipin 1
**LRP5**	LDL receptor related protein 5
**LRRC15**	leucine rich repeat containing 15
**LRRFIP2**	leucine rich repeat (in FLII) interacting protein 2
**LTA**	lymphotoxin alpha
**LYG**	lysozyme G
**LYN**	LYN proto-oncogene, Src family tyrosine kinase
**LYST**	lysosomal trafficking regulator

**LYZC**	lysozyme C
**M17**	IL-6 subfamily member m17
**MAEA**	macrophage erythroblast attacher
**MAF1**	MAF1 homolog, negative regulator of RNA polymerase III
**MALT1**	MALT1 paracaspase
**MAP2K1**	mitogen-activated protein kinase kinase 1
**MAPK1**	mitogen-activated protein kinase 1
**MAPK14**	mitogen-activated protein kinase 14
**MAPK2**	mitogen-activated protein kinase 1
**MAPK3**	mitogen-activated protein kinase 3
**MAPK4**	mitogen-activated protein kinase 4
**MAPK6**	mitogen-activated protein kinase 6
**MAPK7**	mitogen-activated protein kinase 7
**MAPKAPK2**	mitogen-activated protein kinase-activated protein kinase 2
**MAPKAPK3**	mitogen-activated protein kinase-activated protein kinase 3
**MARCKS**	myristoylated alanine rich protein kinase C substrate
**MARK1**	microtubule affinity regulating kinase 1
**MASP2**	mannan binding lectin serine peptidase 2
**MAVS**	mitochondrial antiviral signaling protein
**MCM2**	minichromosome maintenance complex component 2
**MED24**	mediator complex subunit 24
**MEF2C**	myocyte enhancer factor 2C
**MEFV**	Mediterranean fever
**MEIS1**	Meis homeobox 1
**MELK**	maternal embryonic leucine zipper kinase
**METAP2**	methionyl aminopeptidase 2
**MFAP2**	microfibrillar associated protein 2
**MFN2**	mitofusin 2
**MIB1**	mindbomb E3 ubiquitin protein ligase 1
**MID2**	midline 2
**MITF**	microphthalmia-associated transcription factor
**MLLT4**	myeloid/lymphoid or mixed-lineage leukemia; translocated to, 4
**MME**	membrane metallo-endopeptidase
**MMP19**	matrix metallopeptidase 19
**MMP2**	matrix metallopeptidase 2
**MMP9**	matrix metallopeptidase 9
**MR1**	major histocompatibility complex, class I-related
**MR2**	major histocompatibility complex, class II-related
**MS**	multiple sclerosis
**MSH2**	mutS homolog 2
**MSH6**	mutS homolog 6
**MSN**	moesin
**MSRB1**	methionine sulfoxide reductase B1
**MTA2**	metastasis associated 1 family member 2
**MTA3**	metastasis associated 1 family member 3
**MTOR**	mechanistic target of rapamycin
**MX1**	MX dynamin like GTPase 1
**MX2**	MX dynamin like GTPase 2
**MX3**	MX dynamin like GTPase 3
**MYD88**	myeloid differentiation primary response 88

**MYEF2**	myelin expression factor 2
**MYL12**	myosin light chain 12
**MYL2**	myosin light chain 2
**MYLK**	myosin light chain kinase
**MYLPF**	myosin light chain, phosphorylatable, fast skeletal muscle
**MYO1E**	myosin IE
**NAF1**	nuclear assembly factor 1 ribonucleoprotein
**NAR1**	cytosolic FE-S cluster assembly factor partial
**NBEA**	neurobeachin
**NBN**	nibrin
**NCF1**	neutrophil cytosolic factor 1
**NCF2**	neutrophil cytosolic factor 2
**NCF4**	neutrophil cytosolic factor 4
**NCK1**	NCK adaptor protein 1
**NCK2**	NCK adaptor protein 2
**NCOR1**	nuclear receptor corepressor 1
**NDRG1**	N-myc downstream regulated 1
**NEDD4**	neural precursor cell expressed, developmentally down-regulated 4, E3 ubiquitin protein ligase
**NFATC1**	nuclear factor of activated T-cells 1
**NFATC3**	nuclear factor of activated T-cells 3
**NFE2L1**	nuclear factor, erythroid 2 like 1
**NFIL3**	nuclear factor, interleukin 3 regulated
**NFKB1**	nuclear factor of kappa light polypeptide gene enhancer in B-cells 1
**NFKBIA**	NFKB inhibitor alpha
**NFKBIE**	NFKB inhibitor epsilon
**NFKBIL1**	NFKB inhibitor like 1
**NFKBIZ**	NFKB inhibitor zeta
**NFYA**	nuclear transcription factor Y subunit alpha
**NFYB**	nuclear transcription factor Y subunit beta
**NFYG**	nuclear transcription factor Y subunit gamma
**NKIRAS2**	NFKB inhibitor interacting Ras-like 2
**NKRF**	NFKB repressing factor
**NLRC3**	NLR family, CARD domain containing 3
**NLRP1**	NLR family, pyrin domain containing 1
**NLRP3**	NLR family, pyrin domain containing 3
**NLRX1**	NLR family member X1
**NOD1**	nucleotide binding oligomerization domain containing 1
**NOP10**	NOP10 ribonucleoprotein
**NOTCH2**	notch 2
**NPC1**	Niemann-Pick disease, type C1
**NPM1**	nucleophosmin (nucleolar phosphoprotein B23, numatrin)
**NSMAF**	neutral sphingomyelinase activation associated factor
**NUMB**	numb homolog (Drosophila)
**OCLN**	occludin
**ORAI1**	ORAI calcium release-activated calcium modulator 1
**OTUD5**	OTU deubiquitinase 5
**P2RX1**	purinergic receptor P2X 1
**P2RY1**	purinergic receptor P2Y1
**PAFAH1B1**	platelet activating factor acetylhydrolase 1b regulatory subunit 1
**PAK1**	p21 protein (Cdc42/Rac)-activated kinase 1

**PAK2**	p21 protein (Cdc42/Rac)-activated kinase 2
**PAK6**	p21 protein (Cdc42/Rac)-activated kinase 6
**PAK7**	p21 protein (Cdc42/Rac)-activated kinase 7
**PAR1**	coagulation factor II thrombin receptor
**PARD3**	par-3 family cell polarity regulator
**PAXIP1**	PAX interacting protein 1
**PBX1**	pre-B-cell leukemia homeobox 1
**PCGF2**	polycomb group ring finger 2
**PCID2**	PCI domain containing 2
**PDCD2**	programmed cell death 2
**PDE1B**	phosphodiesterase 1B
**PDIA3**	protein disulfide isomerase family A member 3
**PDPK1**	3-phosphoinositide dependent protein kinase 1
**PELI2**	pellino E3 ubiquitin protein ligase 2
**PELI1**	pellino E3 ubiquitin protein ligase 1
**PGM3**	phosphoglucomutase 3
**PGLYRP5**	Peptidoglycan recognition protein 5
**PI4K2A**	phosphatidylinositol 4-kinase type 2 alpha
**PICALM**	phosphatidylinositol binding clathrin assembly protein
**PIEZO1**	piezo type mechanosensitive ion channel component 1
**PIGR**	polymeric immunoglobulin receptor
**PIK3AP1**	phosphoinositide-3-kinase adaptor protein 1
**PIK3C2A**	phosphatidylinositol-4-phosphate 3-kinase catalytic subunit type 2 alpha
**PIK3C2G**	phosphatidylinositol-4-phosphate 3-kinase catalytic subunit type 2 gamma
**PIK3CA**	phosphatidylinositol-4,5-bisphosphate 3-kinase catalytic subunit alpha
**PIK3CB**	phosphatidylinositol-4,5-bisphosphate 3-kinase catalytic subunit beta
**PIK3CG**	phosphatidylinositol-4,5-bisphosphate 3-kinase catalytic subunit gamma
**PIK3R2**	phosphoinositide-3-kinase regulatory subunit 2
**PIM1**	Pim-1 proto-oncogene, serine/threonine kinase
**PIP4K2A**	phosphatidylinositol-5-phosphate 4-kinase, type II, alpha
**PIP5K1A**	phosphatidylinositol-4-phosphate 5-kinase, type I, alpha
**PIR**	pirin
**PITX2**	paired like homeodomain 2
**PLA2**	phospholipase A2 group IB
**PLAT**	plasminogen activator, tissue type
**PLAU**	plasminogen activator, urokinase
**PLCB1**	phospholipase C beta 1
**PLCG1**	phospholipase C gamma 1
**PLCG2**	phospholipase C gamma 2
**PLD1**	phospholipase D1
**PLEC**	plectin
**PLEKHA1**	pleckstrin homology domain containing A1
**PMS2**	PMS1 homolog 2, mismatch repair system component
**PODXL**	podocalyxin like
**POLB**	polymerase (DNA) beta
**PPP2R3C**	protein phosphatase 2, regulatory subunit B", gamma
**PPBP**	pro-platelet basic protein
**PPIA**	peptidylprolyl isomerase A
**PPP1CA**	protein phosphatase 1 catalytic subunit alpha
**PPP1R12A**	protein phosphatase 1 regulatory subunit 12A

**PPP3CA**	protein phosphatase 3 catalytic subunit alpha
**PPP3R1**	protein phosphatase 3 regulatory subunit B, alpha
**PRDM9**	PR domain 9
**PRDX3**	peroxiredoxin 3
**PRF1**	perforin 1
**PRG4**	proteoglycan 4
**PRKAR1A**	protein kinase cAMP-dependent type I regulatory subunit alpha
**PRKCA**	protein kinase C alpha
**PRKCB**	protein kinase C beta
**PRKCD**	protein kinase C delta
**PRKCE**	protein kinase C epsilon
**PRKCI**	protein kinase C iota
**PRKD2**	protein kinase D2
**PRKG1**	protein kinase, cGMP-dependent, type I
**PRKRA**	protein activator of interferon induced protein kinase EIF2AK2
**PSAP**	prosaposin
**PSEN2**	presenilin 2
**PSMA1**	proteasome subunit alpha 1
**PSMA2**	proteasome subunit alpha 2
**PSMA7**	proteasome subunit alpha 7
**PSMB6**	proteasome subunit beta 6
**PSMB9**	proteasome subunit beta 9
**PSMC1**	proteasome 26S subunit, ATPase 1
**PSMC2**	proteasome 26S subunit, ATPase 2
**PSMC5**	proteasome 26S subunit, ATPase 5
**PSMD11**	proteasome 26S subunit, non-ATPase 11
**PSMD13**	proteasome 26S subunit, non-ATPase 13
**PSMD14**	proteasome 26S subunit, non-ATPase 14
**PSMD2**	proteasome 26S subunit, non-ATPase 2
**PSMD3**	proteasome 26S subunit, non-ATPase 3
**PSME1**	proteasome activator subunit 1
**PSME2**	proteasome activator subunit 2
**PSME3**	proteasome activator subunit 3
**PSME4**	proteasome activator subunit 4
**PSTPIP1**	proline-serine-threonine phosphatase interacting protein 1
**PTEN**	phosphatase and tensin homolog
**PTGS1**	prostaglandin-endoperoxide synthase 1 (prostaglandin G/H synthase and cyclooxygenase)
**PTK1**	mitogen-activated protein kinase kinase kinase 11
**PTK2**	protein tyrosine kinase 2
**PTK2B**	protein tyrosine kinase 2 beta
**PTK6**	protein tyrosine kinase 6
**PTK7**	protein tyrosine kinase 7 (inactive)
**PTPN11**	protein tyrosine phosphatase, non-receptor type 11
**PTPN6**	protein tyrosine phosphatase, non-receptor type 6
**PTPN9**	protein tyrosine phosphatase, non-receptor type 9
**PTPRC**	protein tyrosine phosphatase, receptor type C
**PVRL4**	nectin cell adhesion molecule 4
**PXN**	paxillin
**PYCARD**	PYD and CARD domain containing
**PYGO1**	pygopus family PHD finger 1

**RAB10**	RAB10, member RAS oncogene family
**RAB27A**	RAB27A, member RAS oncogene family
**RAB3D**	RAB3D, member RAS oncogene family
**RAB4B**	RAB4B, member RAS oncogene family
**RAB6A**	RAB6A, member RAS oncogene family
**RAB7A**	RAB7A, member RAS oncogene family
**RAC1**	ras-related C3 botulinum toxin substrate 1 (rho family, small GTP binding protein Rac1)
**RAC2**	ras-related C3 botulinum toxin substrate 2 (rho family, small GTP binding protein Rac2)
**RAF1**	Raf-1 proto-oncogene, serine/threonine kinase
**RALB**	v-ral simian leukemia viral oncogene homolog B
**RAP1A**	RAP1A, member of RAS oncogene family
**RAP1B**	RAP1B, member of RAS oncogene family
**RAPGEF4**	Rap guanine nucleotide exchange factor 4
**RASGRP1**	RAS guanyl releasing protein 1
**RASGRP2**	RAS guanyl releasing protein 2
**RBPJ**	recombination signal binding protein for immunoglobulin kappa J region
**RBX1**	ring-box 1, E3 ubiquitin protein ligase
**RCOR1**	REST corepressor 1
**RIG-I**	Retinoic acid-inducible gene-I
**RECK**	reversion inducing cysteine rich protein with kazal motifs
**RELA**	v-rel avian reticuloendotheliosis viral oncogene homolog A
**REST**	RE1 silencing transcription factor
**RFXANK**	regulatory factor X associated ankyrin containing protein
**RGS18**	regulator of G-protein signaling 18
**RHOA**	ras homolog family member A
**RHOC**	ras homolog family member C
**RIPK2**	receptor interacting serine/threonine kinase 2
**RIPK3**	receptor interacting serine/threonine kinase 3
**RNF168**	ring finger protein 168, E3 ubiquitin protein ligase
**ROCK1**	Rho associated coiled-coil containing protein kinase 1
**ROCK2**	Rho associated coiled-coil containing protein kinase 2
**POLR2E**	Polymerase (RNA) II (DNA Directed) Polypeptide E, 25kDa
**POLR2F**	Polymerase (RNA) II (DNA Directed) Polypeptide F
**POLR2H**	Polymerase (RNA) II (DNA Directed) Polypeptide H
**POLR2K**	Polymerase (RNA) II (DNA Directed) Polypeptide K, 7.0kDa
**POLR2L**	Polymerase (RNA) II (DNA Directed) Polypeptide L, 7.6kDa
**POLR1C**	Polymerase (RNA) I Polypeptide C, 30kDa
**POLR1D**	Polymerase (RNA) I Polypeptide D, 16kDa
**POLR3K**	Polymerase (RNA) III (DNA Directed) Polypeptide K, 12.3 KDa
**POLR3B**	Polymerase (RNA) III (DNA Directed) Polypeptide B
**POLR3C**	Polymerase (RNA) III (DNA Directed) Polypeptide C (62kD)
**POLR3D**	Polymerase (RNA) III (DNA Directed) Polypeptide D, 44kDa
**POLR3E**	Polymerase (RNA) III (DNA Directed) Polypeptide E (80kD)
**POLR3F**	Polymerase (RNA) III (DNA Directed) Polypeptide F, 39 KDa
**POLR3G**	Polymerase (RNA) III (DNA Directed) Polypeptide G (32kD)
**RPL11**	ribosomal protein L11
**RPL22**	ribosomal protein L22
**RPL27**	ribosomal protein L27
**RPL35**	ribosomal protein L35
**RPL39**	ribosomal protein L39

**RPLP1**	ribosomal protein lateral stalk subunit P1
**RPS14**	ribosomal protein S14
**RPS17**	ribosomal protein S17
**RPS27**	ribosomal protein S27
**RPS27A**	ribosomal protein S27a
**RPS29**	ribosomal protein S29
**RPS3**	ribosomal protein S3
**RPS6**	ribosomal protein S6
**RPS6KB1**	ribosomal protein S6 kinase B1
**RSAD2**	radical S-adenosyl methionine domain containing 2
**RUNX1T1**	RUNX1 translocation partner 1
**SAE1**	SUMO1 activating enzyme subunit 1
**SAE2**	ubiquitin like modifier activating enzyme 2
**SART3**	squamous cell carcinoma antigen recognized by T-cells 3
**SBDS**	SBDS ribosome assembly guanine nucleotide exchange factor
**SCARA3**	scavenger receptor class A member 3
**SCARA5**	scavenger receptor class A member 5
**SCARF1**	scavenger receptor class F member 1
**SCARF2**	scavenger receptor class F member 2
**SDF1**	C-X-C motif chemokine ligand 12
**SEC13**	SEC13 homolog, nuclear pore and COPII coat complex component
**SEC23B**	Sec23 homolog B, COPII coat complex component
**SEC61A**	Sec61 translocon alpha subunit
**SEC61G**	Sec61 translocon gamma subunit
**SERPINE1**	serpin peptidase inhibitor, clade E (nexin, plasminogen activator inhibitor type 1), member 1
**SF3A3**	splicing factor 3a subunit 3
**SH2B3**	SH2B adaptor protein 3
**SH2D1A**	SH2 domain containing 1A
**SHC1**	SHC (Src homology 2 domain containing) transforming protein 1
**SIKE1**	suppressor of IKBKE 1
**SIN3A**	SIN3 transcription regulator family member A
**SIX1**	SIX homeobox 1
**SKP1**	S-phase kinase-associated protein 1
**SLC25A38**	solute carrier family 25 member 38
**SLC40A1**	solute carrier family 40 member 1
**SLC46A2**	solute carrier family 46 member 2
**SLC7A8**	solute carrier family 7 member 8
**SLC8A3**	solute carrier family 8 member A3
**SLIT2**	slit guidance ligand 2
**SMAD1**	SMAD family member 1
**SMAD3**	SMAD family member 3
**SMAD5**	SMAD family member 5
**SMARCA4**	SWI/SNF related, matrix associated, actin dependent regulator of chromatin, subfamily a, mem.r 4
**SMOX**	spermine oxidase
**SNAP23**	synaptosome associated protein 23kDa
**SNCA**	synuclein alpha
**SNRK**	SNF related kinase
**SNRNP70**	small nuclear ribonucleoprotein U1 subunit 70
**SNX3**	sorting nexin 3
**SNX5**	sorting nexin 5

**SOCS1**	suppressor of cytokine signaling 1
**SOCS3**	suppressor of cytokine signaling 3
**SOCS5**	suppressor of cytokine signaling 5
**SOCS6**	suppressor of cytokine signaling 6
**SOCS7**	suppressor of cytokine signaling 7
**SOD2**	Superoxide Dismutase 2, Mitochondrial
**SOS1**	SOS Ras/Rac guanine nucleotide exchange factor 1
**SOX4**	SRY-box 4
**SOX6**	SRY-box 6
**SP1**	Sp1 transcription factor
**SP140**	SP140 nuclear body protein
**SP3**	Sp3 transcription factor
**SP4**	Sp4 transcription factor
**SP7**	Sp7 transcription factor
**SP9**	Sp9 transcription factor
**SPA**	IgG-binding protein A
**SPINT1**	serine peptidase inhibitor, Kunitz type 1
**SPNA2**	Spectrin alpha 2
**SPRK1**	SRSF protein kinase 1
**SPRY4**	sprouty RTK signaling antagonist 4
**SPT5**	SPT5 homolog, DSIF elongation factor subunit
**SPTBN1**	spectrin beta, non-erythrocytic 1
**SRC**	SRC proto-oncogene, non-receptor tyrosine kinase
**SRF**	serum response factor
**SRMS**	src-related kinase lacking C-terminal regulatory tyrosine and N-terminal myristylation sites
**SRSF4**	serine/arginine-rich splicing factor 4
**SRSF6**	serine/arginine-rich splicing factor 6
**STAT1**	signal transducer and activator of transcription 1
**STAT3**	signal transducer and activator of transcription 3 (acute-phase response factor)
**STAT5B**	signal transducer and activator of transcription 5B
**STIM1**	stromal interaction molecule 1
**STK3**	serine/threonine kinase 3
**STYK1**	serine/threonine/tyrosine kinase 1
**SUFU**	SUFU negative regulator of hedgehog signaling
**SUGT1**	SGT1 homolog, MIS12 kinetochore complex assembly cochaperone
**SUMO1**	small ubiquitin-like modifier 1
**SUMO2**	small ubiquitin-like modifier 2
**SUMO3**	small ubiquitin-like modifier 3
**SYK**	spleen tyrosine kinase
**SYNCRIP**	synaptotagmin binding, cytoplasmic RNA interacting protein
**TAB2**	TGF-beta activated kinase 1/MAP3K7 binding protein 2
**TAB3**	TGF-beta activated kinase 1/MAP3K7 binding protein 3
**TANK**	TRAF family member associated NFKB activator
**TAP1**	transporter 1, ATP-binding cassette, sub-family B (MDR/TAP)
**TAP2**	transporter 2, ATP-binding cassette, sub-family B (MDR/TAP)
**TAPBP**	TAP binding protein (tapasin)
**TBK1**	TANK binding kinase 1
**TBL2**	transducin (beta)-like 2
**TBX1**	T-box 1
**TBXA2R**	thromboxane A2 receptor

**TC1A**	Transposable element Tc1 transposase
**TCF7**	transcription factor 7 (T-cell specific, HMG-box)
**TEC**	tec protein tyrosine kinase
**EEF1E1**	Eukaryotic translation elongation factor 1 epsilon 1
**TF**	transferrin
**TFE3**	transcription factor binding to IGHM enhancer 3
**TFEB**	transcription factor EB
**TFRC**	transferrin receptor
**TGFB1**	transforming growth factor beta 1
**TGFBR2**	transforming growth factor beta receptor II
**THBD**	thrombomodulin
**TIAM1**	T-cell lymphoma invasion and metastasis 1
**TICAM1**	toll like receptor adaptor molecule 1
**TINAGL1**	tubulointerstitial nephritis antigen like 1
**TLN1**	talin 1
**TLR12**	toll-like receptor 12
**TLR13**	toll-like receptor 13
**TLR18**	toll-like receptor 18
**TLR3**	toll like receptor 3
**TLR8**	toll like receptor 8
**TM7SF2**	Transmembrane 7 Superfamily Member 2
**TMOD3**	tropomodulin 3
**TNFAIP1**	TNF alpha induced protein 1
**TNFAIP2**	TNF alpha induced protein 2
**TNFAIP6**	TNF alpha induced protein 6
**TNFAIP8**	TNF alpha induced protein 8
**TNFRSF10B**	tumor necrosis factor receptor superfamily member 10b
**TNFRSF11A**	tumor necrosis factor receptor superfamily member 11a
**TNFRSF11B**	tumor necrosis factor receptor superfamily member 11b
**TNFRSF12A**	tumor necrosis factor receptor superfamily member 12A
**TNFRSF18**	tumor necrosis factor receptor superfamily member 18
**TNFRSF19**	tumor necrosis factor receptor superfamily member 19
**TNFRSF1A**	tumor necrosis factor receptor superfamily member 1A
**TNFRSF1B**	tumor necrosis factor receptor superfamily member 1B
**TNFRSF21**	tumor necrosis factor receptor superfamily member 21
**TNFRSF27**	ectodysplasin A2 receptor
**TNFRSF4**	tumor necrosis factor receptor superfamily member 4
**TNFRSF5**	CD40 molecule
**TNFRSF6**	Fas cell surface death receptor
**TNFRSF9**	tumor necrosis factor receptor superfamily member 9
**TNFSF10**	tumor necrosis factor superfamily member 10
**TNFSF13**	tumor necrosis factor superfamily member 13
**TNK1**	tyrosine kinase, non-receptor, 1
**TNS4**	tensin 4
**TOLLIP**	toll interacting protein
**TP53**	tumor protein p53
**TRADD**	TNFRSF1A-associated via death domain
**TRAF2**	TNF receptor associated factor 2
**TRAF3**	TNF receptor associated factor 3
**TRIM14**	tripartite motif containing 14

**TRIM16**	tripartite motif containing 16
**TRIM2**	tripartite motif containing 2
**TRIM21**	tripartite motif containing 21
**TRIM25**	tripartite motif containing 25
**TRIM29**	tripartite motif containing 29
**TRIM3**	tripartite motif containing 3
**TRIM33**	tripartite motif containing 33
**TRIM35**	tripartite motif containing 35
**TRIM38**	tripartite motif containing 38
**TRIM39**	tripartite motif containing 39
**TRIM44**	tripartite motif containing 44
**TRIM47**	tripartite motif containing 47
**TRIM55**	tripartite motif containing 55
**TRIM65**	tripartite motif containing 65
**TRIM7**	tripartite motif containing 7
**TRIP6**	thyroid hormone receptor interactor 6
**TSSC4**	tumor suppressing subtransferable candidate 4
**TUBB4A**	tubulin beta 4A class IVa
**TUSC2**	tumor suppressor candidate 2
**TXLNA**	taxilin alpha
**TXN**	thioredoxin
**TYK2**	tyrosine kinase 2
**UBA52**	ubiquitin A-52 residue ribosomal protein fusion product 1
**UBE2A**	ubiquitin conjugating enzyme E2 A
**UBE2B**	ubiquitin conjugating enzyme E2 B
**UBE2D2**	ubiquitin conjugating enzyme E2 D2
**UBE2K**	ubiquitin conjugating enzyme E2 K
**UBE2M**	ubiquitin conjugating enzyme E2 M
**UBE2N**	ubiquitin conjugating enzyme E2 N
**UBE2V1**	ubiquitin conjugating enzyme E2 V1
**UBE2V2**	ubiquitin conjugating enzyme E2 V2
**UNC93B1**	unc-93 homolog B1 (C. elegans)
**VAMP7**	vesicle associated membrane protein 7
**VAMP8**	vesicle associated membrane protein 8
**VASP**	vasodilator-stimulated phosphoprotein
**VAV1**	vav guanine nucleotide exchange factor 1
**VCL**	vinculin
**VDAC2**	voltage dependent anion channel 2
**VEGFAA**	Vascular endothelial growth factor A
**VHL**	von Hippel-Lindau tumor suppressor
**VPRBP**	Vpr (HIV-1) binding protein
**WAS**	Wiskott-Aldrich Syndrome
**WASF2**	WAS protein family member 2
**WDR43**	WD repeat domain 43
**WDR55**	WD repeat domain 55
**XCR1**	X-C motif chemokine receptor 1
**YES1**	YES proto-oncogene 1, Src family tyrosine kinase
**YWHAE**	tyrosine 3-monooxygenase/tryptophan 5-monooxygenase activation protein epsilon
**YWHAG**	tyrosine 3-monooxygenase/tryptophan 5-monooxygenase activation protein gamma
**YWHAH**	tyrosine 3-monooxygenase/tryptophan 5-monooxygenase activation protein eta

**YWHAZ**	tyrosine 3-monooxygenase/tryptophan 5-monooxygenase activation protein zeta
**ZAP70**	zeta chain of T cell receptor associated protein kinase 70kDa
**ZBTB16A**	Zinc finger and BTB domain-containing protein 16
**ZNF214**	Zinc Finger Protein 214
**ZNF271**	Zinc Finger Protein 271
**ZNF574**	Zinc Finger Protein 574
**ZNF850**	Zinc Finger Protein 850
