# Supplementary material for: De novo assembly of the sea trout (Salmo trutta m. trutta) skin transcriptome to identify putative genes involved in the immune response and epidermal mucus secretion
Source: PLoS One. 2017 Feb 17;12(2):e0172282. doi: 10.1371/journal.pone.0172282 (PMC5315281; doi:10.1371/journal.pone.0172282)
Supplement: S7 Table — (PDF) [file pone.0172282.s010.pdf]

S7 Table. Distribution of EST-SSRs based on motif types.

**Motif**	**Motif Length**	**Total Counts**
AC	2	394
AG	2	90
AT	2	73
CT	2	124
GT	2	309
AAT	3	4
ACT	3	7
AGC	3	1
AGT	3	19
ATT	3	9
CAA	3	3
GAG	3	2
GAT	3	3
AAGG	4	1
ACAG	4	17
ACAT	4	17
ACTG	4	2
AGAA	4	2
AGAT	4	4
AGGT	4	1
ATGG	4	1
ATGT	4	13
CAAT	4	1
CAGT	4	1
CGGT	4	1
CTAT	4	1
CTCA	4	1
CTGT	4	7
TCCA	4	1
TCTT	4	3
TTGA	4	1
TTTG	4	1
ACAAA	5	1
AGAGT	5	2
CCTGT	5	1
GAGAG	5	1
